# Experience of health care utilization for inpatient and outpatient services among older adults in India

**DOI:** 10.1016/j.puhip.2024.100541

**Published:** 2024-08-22

**Authors:** Mayanka Ambade, Rockli Kim, S.V. Subramanian

**Affiliations:** a Indian Institute of Technology Mandi, India; b Division of Health Policy and Management, College of Health Science, Korea University, Seoul, South Korea; c Interdisciplinary Program in Precision Public Health, Department of Public Health Sciences, Graduate School of Korea University, Seoul, South Korea; d Harvard Center for Population and Development Studies, 9 Bow Street Cambridge, MA, 02138, USA; e Department of Social and Behavioral Sciences, Harvard T. H. Chan School of Public Health, Boston, MA, USA

**Keywords:** Patient experience, Older adults, Quality of healthcare, India

## Abstract

**Background:**

Patient experiences have not been documented at all India level among older adults for inpatient and outpatient services. We provide all-India and sub national estimates on six domains of patient experience, namely: waiting time, respectful treatment, clarity of explanation provided, privacy during consultation, treated by provider of choice, and cleanliness of facility.

**Methods:**

Unit records of adults aged 45 years and above for their inpatient (n = 4330) or outpatient (n = 33,724) service use were assessed from the Longitudinal Ageing Survey of India (LASI), conducted in 2017-18. We identified patient experience as negative if the respondent rated it as either “Bad” or “Very Bad” on a five-point Likert scale. We computed proportion of negative experience by socio-economic status, geographic location, and type of healthcare facilities. We used binary logistic regression to estimate predictors of negative patient experience, and a three-level logistic regression model to partition the total geographic variation of patient experiences.

**Findings:**

Most individuals rated their experience in all six domains as “Good”. Negative experiences were higher among patients who used public facilities, specifically for waiting time and cleanliness of facility. Among inpatients, the higher-than-average negative experience was noted in the north and northwest, while among outpatients, it was higher in the northeast. The largest geographic variation in negative patient experience was attributable to the villages/CEBs for all domains in outpatient services and three domains of inpatient services, whereas states accounted for the other three inpatient domains.

**Interpretation:**

Majority of older adults rated their experience of healthcare use positively, but less for public health facilities.

## 1 Introduction

Currently, health systems in Low-and-Middle Income Countries (LMICs) often provide inadequate quality of care, resulting in 60 % of institutional deaths being attributed to poor quality [1]. A significant proportion of patients in LMICs report negative health service experiences, particularly affecting vulnerable groups such as the poor and less educated. This substandard care contributes to unnecessary suffering, persistent symptoms, and undermines trust in the health system. Despite evidence suggesting that improving care quality is more crucial for reducing mortality than insufficient access, LMIC health systems tend to prioritize expanding services over simultaneous improvements in quality. For instance, India's National Health Policy (2017) aimed to deliver high-quality, affordable healthcare, yet its primary focus remained on expanding health infrastructure coverage [2].

Meaningful advancement in quality can be achieved through accountability via regular measurement. However, current surveys and administrative data in India focus on health infrastructure to judge the quality of health care centres [3,4]. Such input-based quality measures provide narrow insights and leave large gaps in our understanding of user experience or their confidence in the system [1]. The World Health Organisation [5] along with two most influential quality of care reports, one by the Institute of Medicine [6] and the other by the Lancet Global Health Commission on High Quality Health Systems [1], consider patient-centeredness as primary domain of quality along with correctness of the prognosis. Patient-centred measures, such as patient-related experience measures (PREM), assess aspects valued by patients [7]—like timely appointments and respectful treatment. PREM are reliable indicators across health systems [8,9], linked to improved clinical effectiveness, medication adherence, and confidence in healthcare [[10], [11], [12], [13]]. OECD countries, including the USA and UK, are integrating patient-centred measures into national frameworks to enhance healthcare quality assessment [14,15].

India's healthcare system features public, private, and community providers across all levels, with a predominant reliance on private healthcare funded out of pocket by most individuals. The public sector offers subsidized care, and private providers, concentrated in urban areas, also deliver primary services in rural regions. Despite some private health insurance, national policies struggle with coverage and eligibility issues, limiting their effectiveness. While state governments regulate the public sector, private providers operate largely independently, albeit expected to meet certain standards. This setup results in significant market failures, and given low regulation, the quality of healthcare may get compromised as suggested by some empirical evidences [[16], [17], [18]]. Studies have found widespread low-quality primary care across Indian districts, deficiencies in treating childhood diarrhoea and pneumonia, and significant under-detection of high-risk pregnancies among community health workers [[16], [17], [18]].

Further, given that health is a state subject, and districts and village become the unit of execution, quality of care is highly variable across geographic regions [16]. Thus, a comprehensive assessment of healthcare quality, for inpatients and outpatients, at national and subnational levels becomes essential, especially using PREM measures. Also, such an assessment for older adults gains larger significance, given that with rapid ageing, older adults will dominate the epidemiological landscape of India in the coming years; and learning about their experiences is crucial. However, systematic nationally representative evidence on patient experience (or PREM) is lacking in India, thus limiting a comprehensive assessment of its health system quality. At best, one can find state-level or localised patient experience sample surveys, sometimes restricted to specific medical conditions such as cancer or maternity care [[19], [20], [21], [22], [23], [24]]. Even the internet-based survey of 12 low-and-middle income countries conducted by the Lancet Global Health Commission provides little idea about the sub-national performance of health facilities in India [1]. Therefore, using the patient experience data from the nationally representative Longitudinal Ageing Survey of India (LASI) that collects information on population aged 45 years or above and their spouses, we aim to provide a comprehensive assessment of quality of healthcare service as perceived by the users for both inpatient and outpatient services, for all India as well as states [25].

## 2 Materials and methods

### 2.1 Data source and survey design

This cross-sectional study follows the Strengthening the Reporting of Observational Studies in Epidemiology (STROBE) reporting guidelines. We used unit level data of individuals aged 45 years and above from the first wave of Longitudinal Ageing Study in India (LASI) conducted in 2017-18 [25]. Our team did not have access to any identifiers linked to the data, and thus did not meet the regulatory definition of human participation research, thereby exempting our study from a full institutional review as per Harvard Longwood Campus Institutional Review Board's (IRB) self-determination guidelines.

LASI used stratified, multistage cluster sampling to select households. All members of the household aged 45 years and above and their spouse were interviewed in depth. The survey had a minimum sample size of 1000 individuals, with larger samples in states with larger populations, and a response rate of 87.3 % for individuals. The survey is representative at state levels. Further details of the survey can be found in their national report [25].

### 2.2 Study population

We performed a complete case analysis for all older adults aged 45 years, i.e., no missing information for any of the patient experience questions. This sample was different for those who used inpatient and outpatient services. The inpatient (and outpatient) analyses were restricted to the individuals who reported being hospitalized (or used outpatient facility) in the past 12 months. Therefore, we used the ‘type of facility’ information for the latest hospital (or OPD) use as patient experiences were captured only for the same.

### 2.3 Measures of patient experience

In LASI, all participants who reported use of inpatient or outpatient services in the past 12 months were asked about their experience of last hospitalisation or OPD consultation, respectively. The following questions captured the patient experience for hospitalisation [and OPD]: “For your last hospitalisation or stay at a long-term care facility [or last visit to a hospital or health care facility], how would you rate the following: (a) … your experience about the length of time you waited before being attended to; (b) … your experience of being treated respectfully; (c) … your experience on how clearly health care providers explained things to you, (d) … your experience of the way the health care staff ensured that you could talk privately to providers, (e) … your experience of getting a health care provider of your choice, and (f) … your experience about the cleanliness in the health facility”. The respondents were asked to rate their experience on a five-point Likert scale that took the values of 1 for “Very Good”, 2 for “Good”, 3 for “Neither Good nor Bad”, 4 for “Bad”, and 5 for “Very Bad”. Our outcome variables are based on the abovementioned six questions, and we refer to them, respectively, as (a) Waiting time, (b) Respectful treatment, (c) Clarity of explanation provided, (d) Privacy during consultation, (e) Treated by the provider of choice and (f) Cleanliness of facility. We identified individuals with a negative experience of healthcare use for each of the six items if they reported their experience as ‘bad’ or ‘very bad’ on the five-point Likert Scale.

### 2.4 Covariates

The covariates were included in the study based on literature [26,27]. We examined the variation in each of the six items for inpatients and outpatients across states and union territories of India. We also estimated the same across type of facility (public, private and other) and place of residence (rural and urban). Further, we have calculated the percentage of respondents who reported a negative experience (rated as ‘bad’ or ‘very bad’) by place of residence, type of facility, gender (male/female), age (45–54 years, 55 to 64, 65–74 years, and 75+), marital status (currently married, currently unmarried), religion (Hindu, Muslim, others), caste (Scheduled Castes, Scheduled tribes, Other Backward Classes, Others), education (illiterate, less than 5 years completed, 5–9 years completed, 10 years or more completed), and expenditure quintiles (lowest, low, middle, high, highest), working status (currently working, ever worked but currently not working, never worked), reason for hospitalisation (sickness/illness, injury/accident, other).

### 2.5 Statistical analysis

Primarily, our study is a univariate, bi-variate, and multivariate analysis of prevalence of negative patient experience on the six domains across geographic and socio-economic characteristics of patients. We applied sampling weights for all analysis to account for the multistage stratified design of LASI. We reported the percentage of negative experiences by the covariates and 36 states/union territories of India. Binary logistic regression was used to assess the association with individual socioeconomic characteristics. While the predictors were based on literature, we also undertook a correlation analysis before including them in the model (Table S1).

We employed a three-level binary logistic regression model to partition the total geographic variation in all six dimensions of patient experiences for inpatients and outpatients separately. The response variable which initially had five categories was regrouped into two categories of “negative” (that includes only “bad” and “very bad” experiences and identified as 1) and “nonnegative” (identified as 0) patient experience. By doing so, we aimed to differentiate those with clear negative experiences from those who might have given moderate ratings for various reasons such as social desirability or confusion about the question. The LASI data is structured such that each individual was nested in a household, then a village (if rural area) and a Census Enumeration Block (CEB) within a ward (if urban area), followed by the Taluka (sub-district) and the 36 states/union territories as the last stage [23]. Since the information of Taluka (sub-district) has not been released by LASI, we restricted the decomposition of variance to three levels, namely, household (Level-2), Village/CEB (Level-3) and States/UTs (Level-4). The mathematical form of the three-stage logistic regression is as follows-

$${\log\;I\left(\pi_{ijkl}\right)=\beta_{0}+BX}_{ijkl}+g_{0l}+f_{0kl}+v_{0jkl}$$

Which estimates the probability of a negative experience for individual ‘i’ in household ‘j’, village/CEB ‘k’ and state ‘l’. In this model, $g_{0l},f_{0kl}\;and\;v_{0ijk}$ indicate the residual difference at the state, village/CEB, and household levels respectively.

In this model, we considered residual differences at each level: state, village/CEB, and household. We assumed these residuals followed a normal distribution with specific variances, representing differences between states, villages/CEBs, and households. To understand how much each geographic unit contributed to the overall variability in patient experiences, we calculated the Variance Partitioning Coefficient (VPC). This coefficient assesses the significance of each geographic unit by comparing its variance to the total variance across all levels.

We conducted this analysis using the *runmlwin* in Stata-18 software [28]. We employed Monte Carlo Markov Chain (MCMC) methods with a Gibbs sampler to estimate parameters, with specific settings for the starting values, burn-in cycles, and monitoring iterations. Monte Carlo Markov Chain with Gibbs Sampler is a statistical method to simulate and explore complex probability distributions by generating samples from them. This process is continued till all samples closely resemble samples from the true distribution. This is used to estimate uncertainty associated with the estimates we generated.

## 3 Results

In LASI, among 72,270 observations, 4781 were hospitalized and 37,494 used outpatient services in the past year. After excluding individuals under 45 years old and those with missing information, 4330 had complete hospitalisation records and 33,724 had complete outpatient records. Both groups predominantly consisted of individuals under 65 years old, with higher proportions of females, married individuals, and lower education levels. Hospitalized individuals were more likely to be in higher income quintiles, while outpatients were predominantly in lower income quintiles (Table 1).

**Table 1:** Percentage distribution of study sample by patient characteristics of older adults aged 45 years and above, India, 2017-18.

	Inpatient		Outpatient	
	Sample	%	Sample	%
**Age**				
45–54	1286	28.9	11,665	32.7
55–64	1351	30.5	10,483	30.6
65–74	1132	27.0	8098	25.5
75+	561	13.6	3478	11.2
**Gender**				
Male	2103	48.3	14,823	44.0
Female	2227	51.7	18,901	56.0
**Marital Status**				
Currently married	3216	73.0	24,829	73.1
Currently unmarried	1114	27.0	8895	27.0
**Education**				
Illiterate	1966	51.0	15,829	51.1
Less than 5 years	575	13.0	3896	11.5
5–9 years completed	1033	22.3	7710	20.9
10 years or more completed	756	13.7	6289	16.5
**Work status**				
Currently working	1560	34.5	14,637	45.3
Ever worked but currently not working	1704	43.1	9711	29.8
Never worked	1066	22.4	9376	24.8
**Expenditure Quintile**				
Lowest	538	13.3	5753	18.6
Lowest	686	16.5	6680	21.3
Middle	789	19.1	6812	20.7
High	946	20.8	7198	20.4
Highest	1371	30.4	7281	19.0
**Religion**				
Hindu	3216	78.7	24,292	81.1
Muslim	519	14.4	4615	12.7
Others	592	6.8	3759	6.0
**Caste**				
Scheduled Castes	759	20.9	6122	20.2
Scheduled Tribes	633	7.1	3818	6.7
Other backward castes	1676	45.0	13,429	44.6
Others	1262	27.1	10,355	28.5
**Place of residence**				
Rural	2804	67.6	21,735	70.6
Urban	1526	32.4	11,989	29.4
**Facility type**				
Public	1858	36.2	10,252	23.0
Private	2357	61.0	19,285	64.0
Other	115	2.8	4187	13.6
**Reason for hospitalisation**				
Sickness/Illness	3858	89.7	24,682	75.6
Injury/Accident	451	9.9	1091	3.1
Other	21	0.4	7951	21.4
Total	4330	100	33,724	100

Using percentage distribution of reported categories of experience, we estimated that among inpatients in the past 12 months, the majority rated their experience of hospital stay as ‘good’ across all six patient experience items (Table 2) - with their percentage share ranging from 48.2 % for waiting time to 40.4 % for cleanliness of facility. The percentage of older adults who reported a negative experience ranged from 4.9 % (bad- 4.0 % and very bad- 0.9 %) for cleanliness of facility to 3.8 % (3.0%- bad, 0.8%- very bad) for clarity of explanation provided. Among outpatients, most rated their experience as ‘good’ for all six items that ranged from 48.2 % for respectful treatment to 45.5 % for privacy during consultation. The percentage of older adults who reported a negative experience ranged from 4.6 % (bad- 4.0 %, very bad- 0.6 %) for waiting time to 2.2 % (bad- 1.9 %, very bad- 0.3 %) for receiving respectful treatment. Public facilities were consistently rated with the highest negative experience across all six domains for both outpatients and inpatients, except for rural inpatient services, where highest negative rating was given to ‘Other’ facility type (Fig. S1). Within the public facilities, highest negative rating was noted for cleanliness of facility for hospitalisation at 9.1 % (8.1 % - ‘bad’ and 1.4%-‘very bad’), and waiting time for OPD services at 9.5 % (8.2%- ‘bad’ and 1.3%-‘very bad’) (Table 2). Within private facilities, the highest negative rating was noted for waiting time for both inpatients and outpatients at 3.3 % and 3.5 % respectively. Moderate ratings for inpatient and outpatient service use varied between 20 and 25 % overall across all six domains; with little variation across these domains. However, more than 25 % of older adults rated moderately on all six domains for both inpatient and outpatient services in public facilities; while this percentage was closer to 20 % or lower in private facilities.

**Table 2:** Percentage distribution of ratings for each of the six domains of patient experience for inpatient and outpatient service use among older adults aged 45 years and above, Inda, 2017-18.

	Inpatient						Outpatient					
	Waiting time	Respectful treatment	Clarity of explanation provided	Privacy during consultation	Treated by provider of choice	Cleanliness of facility	Waiting time	Respectful treatment	Clarity of explanation provided	Privacy during consultation	Treated by provider of choice	Cleanliness of facility
**Overall**												
Very good	26.4	27.2	32.3	28.9	28.2	33.6	22.2	25.0	27.3	26.7	26.6	28.5
Good	46.8	47.0	42.5	41.6	44.0	38.6	48.1	48.2	46.7	45.5	47.0	46.4
Moderate	22.1	22.0	21.4	24.8	23.1	22.9	25.1	24.6	23.1	24.5	23.3	22.4
Bad	3.6	3.2	3.0	3.6	3.9	4.0	4.0	1.9	2.5	2.8	2.7	2.3
Very Bad	1.1	0.7	0.8	1.1	0.9	0.9	0.6	0.3	0.5	0.5	0.5	0.5

**Public facility**												
Very good	18.3	19.4	20.9	20.1	19.1	23.9	15.0	18.6	19.8	18.3	18.0	19.5
Good	46.4	44.3	46.5	44.5	41.6	40.0	44.2	46.1	45.6	44.3	45.3	45.1
Moderate	29.1	30.6	27.6	27.6	31.7	26.6	31.4	31.2	29.2	31.1	30.6	29.3
Bad	4.8	5.1	4.3	6.5	6.4	8.1	8.2	3.6	4.3	5.6	5.1	5.0
Very Bad	1.4	0.7	0.7	1.4	1.2	1.4	1.3	0.6	1.1	0.7	0.9	1.2

**Private facility**												
Very good	30.5	31.1	38.6	33.5	33.0	38.7	24.2	27.4	30.2	29.5	29.9	32.4
Good	48.2	49.5	41.2	40.6	46.2	38.7	49.1	49.7	47.7	46.6	48.2	47.2
Moderate	18.0	16.8	17.5	23.3	18.0	20.6	23.2	21.3	19.9	21.5	19.5	18.6
Bad	2.9	2.0	2.1	1.9	2.3	1.6	3.0	1.4	1.9	2.1	2.0	1.4
Very Bad	0.5	0.5	0.6	0.7	0.5	0.4	0.5	0.3	0.3	0.4	0.4	0.4

**Other facility**												
Very good	43.4	43.1	43.3	41.9	40.2	46.8	24.9	24.8	26.5	27.8	25.3	25.1
Good	23.5	25.9	19.1	26.3	24.9	20.0	49.6	45.0	43.6	42.4	44.0	44.8
Moderate	23.6	22.8	26.0	22.1	24.1	23.5	23.6	28.8	27.7	27.6	28.8	28.2
Bad	1.5	3.4	6.8	4.1	6.1	5.0	1.7	1.2	2.1	1.7	1.4	1.6
Very Bad	8.1	4.8	4.8	5.6	4.8	4.8	0.2	0.2	0.2	0.4	0.5	0.3

### 3.1 Socio-economic and geographic variation

A nominal difference was noted in patient experience on all six domains by gender, marital status, caste groups, and place of residence; with a slightly higher negative experience noted among males, those currently unmarried, and the rural residents for inpatients and outpatients alike (Tables S2 and S3). With age we note a monotonous increase, while with education we observe a decline in reported negative experiences for all six domains and among inpatient sand outpatients alike. A steeper decline is noted with income, for both inpatients and outpatients as seen in the case of cleanliness of facility among inpatients.

In most states, fewer than 5 % of older adults reported negative experiences across all six domains for both inpatient and outpatient care (Table S4 and S5) . Among inpatients, Jammu and Kashmir had the highest negative ratings for waiting time (18.9 %) and respectful treatment (7.4 %), while Lakshadweep had the lowest at 0 %. Haryana reported the highest negative experiences for clarity of explanation (8.6 %), and provider of choice (12.2 %), Sikkim had highest for privacy during consultation (10.3 %). Tripura had the highest negative rating for cleanliness of the facility (12.3 %), followed by West Bengal at 11.4 %. Among outpatients (Table S5), Andaman and Nicobar had the highest negative experience (30.7 %) for waiting time, Madhya Pradesh was highest for respectful treatment (7.0 %), Chattisgarh for cleanliness of facility (9.7 %), and Madhya Pradesh again for clarity of explanation (9.0 %); Haryana was highest for privacy during consultation (7.5 %), and treatment by provider of choice (7.8 %).

Certain states such as Assam, Gujarat, Himachal Pradesh, Kerala Maharashtra, and Mizoram have received lower than average negative ratings on at least five domains for both inpatient and outpatient services. Whereas, certain states such as Tamil Nadu perform better for inpatient, but not so much for outpatient services. Similarly, some states such as Punjab do better for outpatient but have higher than average negative experiences for inpatient services.

### 3.2 Predictors of negative patient experience

Socioeconomic and demographic factors account for 6–11 % of patient experience variation. Facility type is crucial, with private facility users reporting fewer negative experiences across all domains. For example, among inpatients, the odds of reporting negative experience for cleanliness in private facilities is 0.28 (95 % CI: 0.2–0.4; p < 0.001) compared to public (Table 3). Urban residents report higher negative experiences for clarity of explanations (AOR: 1.59; 95 % CI: 1.06–2.39; p < 0.001), and females are half as likely as males to report negative experience for waiting time (AOR: 0.59; 95 % CI: 0.39–0.89; p < 0.001). Education, caste, and income show associations with all the six domains. Higher income correlates with fewer negative experience for cleanliness, and lower castes report more privacy issues during consultations (AOR for Scheduled Tribes vs. Scheduled Castes: 2.3; 95 % CI: 1.27–4.28; p < 0.001). Education, employment status, and facility type predict moderate ratings across all domains. Those with 10+ years of education are less likely to give moderate ratings (AOR: 0.66; 95 % CI: 0.50–0.87; p < 0.001), just as those who are never-employed (AOR: 1.35; 95 % CI: 1.06–1.72; p < 0.01). Variation in patient experience is mostly due to villages for waiting time (73.4 %) and cleanliness (58.8 %), with state-level variations significant for other domains (Table 5).

**Table 3:** Binary logistic regression results (Adjusted Odds Ratio) for association between socio-economic characteristics of older adult inpatients with their negative experience of hospitalisation on six domains of healthcare, India, 2017-18.

VARIABLES	Waiting time	Respectful treatment	Clarity of explanation provided	Privacy during consultation	Provider of Choice	Cleanliness of facility
**Age**						
45-54®						
55–64	0.96	1.17	0.96	0.75	1.01	1.19
	(0.64–1.44)	(0.71–1.92)	(0.59–1.54)	(0.50–1.12)	(0.68–1.50)	(0.79–1.80)
65–74	1.24	1.46	1.23	1.21	0.99	1.3
	(0.79–1.94)	(0.85–2.49)	(0.75–2.01)	(0.78–1.88)	(0.62–1.58)	(0.82–2.05)
75+	1.27	1.44	1.07	1.16	0.95	1.45
	(0.72–2.23)	(0.75–2.76)	(0.59–1.92)	(0.67–2.03)	(0.53–1.72)	(0.84–2.48)
**Gender**						
Male®						
Female	0.59**	0.73	0.84	0.97	1.14	0.82
	(0.39–0.89)	(0.48–1.13)	(0.55–1.30)	(0.67–1.42)	(0.75–1.74)	(0.56–1.22)
**Marital Status**						
Currently married®						
Currently unmarried	1.13	1.21	1.07	1.04	1.02	1.42*
	(0.79–1.63)	(0.81–1.82)	(0.71–1.63)	(0.72–1.52)	(0.69–1.50)	(0.99–2.02)
**Education**						
Illiterate®						
Less than 5 years	1.12	1.24	1.42	1.06	1.07	1.16
	(0.71–1.74)	(0.73–2.09)	(0.89–2.28)	(0.67–1.69)	(0.62–1.88)	(0.71–1.90)
5–9 years completed	1.04	0.72	0.61**	0.82	0.96	1.1
	(0.71–1.54)	(0.43–1.19)	(0.38–0.99)	(0.53–1.26)	(0.62–1.49)	(0.72–1.66)
10 years or more completed	0.87	0.56*	0.65	0.61	1	1.04
	(0.52–1.46)	(0.29–1.09)	(0.36–1.19)	(0.34–1.10)	(0.60–1.68)	(0.60–1.80)
**Work status**						
Currently working®						
Ever worked but currently not working	0.93	1	1.41	1.1	1.14	0.96
	(0.63–1.36)	(0.62–1.60)	(0.91–2.18)	(0.73–1.64)	(0.76–1.71)	(0.65–1.43)
Never worked	1.25	1.19	1	0.99	1.05	1.2
	(0.77–2.03)	(0.68–2.10)	(0.57–1.73)	(0.62–1.60)	(0.64–1.72)	(0.75–1.91)
**Expenditure Quintile**						
Lowest®						
Lowest	1.05	0.94	0.85	1.06	0.66	0.73
	(0.63–1.75)	(0.53–1.65)	(0.49–1.47)	(0.62–1.83)	(0.39–1.12)	(0.44–1.20)
Middle	0.84	1	0.61	1.18	0.67	0.7
	(0.50–1.42)	(0.55–1.81)	(0.34–1.11)	(0.67–2.05)	(0.40–1.14)	(0.42–1.16)
High	0.69	0.9	0.98	0.9	0.71	0.57**
	(0.41–1.17)	(0.50–1.62)	(0.55–1.72)	(0.53–1.55)	(0.42–1.19)	(0.34–0.94)
Highest	0.7	0.55*	0.63	0.8	0.59**	0.49***
	(0.43–1.15)	(0.30–1.01)	(0.35–1.11)	(0.46–1.41)	(0.35–0.99)	(0.30–0.81)
**Religion**						
Hindu®						
Muslim	0.99	0.78	0.8	0.72	0.89	0.78
	(0.59–1.67)	(0.43–1.43)	(0.44–1.44)	(0.38–1.38)	(0.50–1.59)	(0.44–1.39)
Others	1.21	1.38	1.73*	1.21	0.76	1.87**
	(0.74–1.98)	(0.72–2.65)	(0.92–3.25)	(0.69–2.12)	(0.37–1.54)	(1.11–3.15)
**Caste**						
Scheduled Castes®						
Scheduled Tribes	0.92	1.71	2.17**	2.33***	1.29	1.4
	(0.49–1.72)	(0.83–3.51)	(1.10–4.29)	(1.27–4.28)	(0.65–2.56)	(0.78–2.51)
Other backward castes	1.03	1.35	1.21	1.54*	1.2	0.94
	(0.65–1.63)	(0.78–2.32)	(0.71–2.05)	(0.96–2.45)	(0.75–1.93)	(0.61–1.46)
Others	1.06	1.31	1.19	1.39	1.13	1.06
	(0.64–1.75)	(0.70–2.46)	(0.65–2.19)	(0.81–2.38)	(0.67–1.92)	(0.65–1.72)
**Place of residence**						
Rural®						
Urban	0.99	1.4	1.59**	1.2	0.96	1.01
	(0.68–1.44)	(0.91–2.15)	(1.06–2.39)	(0.79–1.82)	(0.65–1.41)	(0.70–1.46)
**Facility type**						
Public®						
Private	0.50***	0.41***	0.58***	0.45***	0.36***	0.28***
	(0.36–0.69)	(0.28–0.61)	(0.40–0.84)	(0.32–0.62)	(0.25–0.51)	(0.20–0.40)
Other	0.61	0.66	1.8	0.71	0.88	0.63
	(0.22–1.72)	(0.20–2.16)	(0.78–4.12)	(0.27–1.87)	(0.38–2.06)	(0.22–1.79)
**Reason for hospitalisation**						
Sickness/Illness®						
Injury/Accident	1.11	1.16	1.13	0.94	1.26	0.97
	(0.10–6.34)	(0.13–11.38)	(0.13–14.38)	(0.10–7.77)	(0.13–8.46)	(0.11–9.92)
Other	0.81	1.22	1.38	0.88	1.04	1.06
	(0.10–6.34)	(0.13–11.38)	(0.13–14.38)	(0.10–7.77)	(0.13–8.46)	(0.11–9.92)

**Constant**	0.25**	0.05***	0.03***	0.08***	0.09***	0.10***
	(0.09–0.73)	(0.01–0.19)	(0.01–0.18)	(0.02–0.30)	(0.02–0.31)	(0.03–0.35)

**Note:** p-values are represented as: ***<0.001; **<0.01; *<0.05.

**Table 4:** Binary logistic regression results (Adjusted Odds Ratio) for the association between socio-economic characteristics of older adult outpatients with their experience of outpatient service use in six domains of healthcare, India, 2017-18.

VARIABLES	Waiting time	Respectful treatment	Clarity of explanation provided	Privacy during consultation	Provider of Choice	Cleanliness of facility
**Age**						
45-54®						
55–64	0.95	0.97	0.97	0.87*	0.86**	0.88
	(0.83–1.08)	(0.80–1.18)	(0.81–1.15)	(0.75–1.02)	(0.73–1.00)	(0.74–1.05)
65–74	0.93	1.00	0.92	0.98	0.86	1.00
	(0.80–1.08)	(0.80–1.23)	(0.75–1.12)	(0.82–1.16)	(0.72–1.03)	(0.83–1.21)
75+	0.87	0.91	0.88	0.92	0.73**	0.95
	(0.70–1.07)	(0.69–1.22)	(0.68–1.12)	(0.72–1.17)	(0.57–0.94)	(0.73–1.22)
**Gender**						
Male®						
Female	1.03	0.92	0.99	0.85**	0.92	0.88
	(0.90–1.18)	(0.75–1.13)	(0.84–1.18)	(0.72–1.00)	(0.78–1.09)	(0.74–1.05)
**Marital Status**						
Currently married®						
Currently unmarried	0.97	1.14	1.05	0.99	0.95	1.10
	(0.85–1.10)	(0.95–1.37)	(0.90–1.24)	(0.85–1.16)	(0.81–1.11)	(0.93–1.31)
**Education**						
Illiterate®						
Less than 5 years	0.98	0.90	0.80*	0.84*	0.86	0.79*
	(0.82–1.17)	(0.69–1.16)	(0.62–1.02)	(0.68–1.03)	(0.69–1.08)	(0.62–1.00)
5–9 years completed	0.93	0.71***	0.95	0.81**	0.86	0.79**
	(0.81–1.07)	(0.56–0.89)	(0.79–1.13)	(0.67–0.96)	(0.72–1.03)	(0.64–0.97)
10 years or more completed	0.86	0.58***	0.64***	0.59***	0.62***	0.83
	(0.72–1.03)	(0.44–0.77)	(0.50–0.82)	(0.47–0.75)	(0.49–0.78)	(0.65–1.07)
**Work status**						
Currently working®						
Ever worked but currently not working	1.1	1.32***	1.30***	1.24**	1.27***	1.07
	(0.97–1.27)	(1.08–1.62)	(1.10–1.53)	(1.05–1.46)	(1.08–1.50)	(0.90–1.28)
Never worked	1.14	1.37***	1.19	1.22*	1.19*	1.29**
	(0.97–1.35)	(1.07–1.75)	(0.96–1.47)	(0.99–1.49)	(0.97–1.47)	(1.05–1.60)
**Expenditure Quintile**						
Lowest®						
Lowest	0.91	0.97	0.89	1.00	1.01	0.82*
	(0.77–1.09)	(0.76–1.23)	(0.71–1.10)	(0.81–1.22)	(0.83–1.24)	(0.66–1.02)
Middle	0.9	1.14	0.92	0.91	1.04	0.99
	(0.75–1.08)	(0.89–1.46)	(0.74–1.14)	(0.74–1.12)	(0.84–1.29)	(0.80–1.22)
High	0.87	0.85	0.88	0.88	0.90	0.87
	(0.72–1.05)	(0.65–1.11)	(0.70–1.10)	(0.70–1.09)	(0.72–1.12)	(0.69–1.09)
Highest	0.74***	0.80*	0.84	0.91	0.88	0.68***
	(0.61–0.91)	(0.60–1.07)	(0.66–1.08)	(0.72–1.16)	(0.69–1.13)	(0.53–0.88)
**Religion**						
Hindu®						
Muslim	0.93	0.86	0.81	0.80	0.85	0.77*
	(0.75–1.15)	(0.61–1.19)	(0.59–1.11)	(0.59–1.10)	(0.64–1.12)	(0.56–1.07)
Others	1.01	1.01	0.86	0.85	1.07	1.12
	(0.78–1.31)	(0.68–1.49)	(0.62–1.21)	(0.63–1.14)	(0.80–1.43)	(0.83–1.50)
**Caste**						
Scheduled Castes®						
Scheduled Tribes	1.06	1.17	1.14	1.09	1.00	0.90
	(0.79–1.42)	(0.84–1.63)	(0.83–1.58)	(0.84–1.41)	(0.75–1.34)	(0.67–1.19)
Other backward castes	1.1	1.01	1.01	1.05	0.95	1.04
	(0.92–1.32)	(0.80–1.26)	(0.83–1.23)	(0.86–1.28)	(0.78–1.15)	(0.85–1.27)
Others	1.02	1.13	1.03	0.89	0.92	0.94
	(0.84–1.24)	(0.88–1.46)	(0.81–1.30)	(0.70–1.12)	(0.74–1.15)	(0.75–1.19)
**Place of residence**						
Rural®						
Urban	1.00	1.27**	0.92	1.00	0.94	0.91
	(0.85–1.18)	(1.03–1.55)	(0.76–1.12)	(0.83–1.21)	(0.79–1.13)	(0.75–1.11)
**Facility type**						
Public®						
Private	0.35***	0.34***	0.39***	0.35***	0.35***	0.26***
	(0.31–0.40)	(0.29–0.41)	(0.33–0.46)	(0.30–0.41)	(0.29–0.41)	(0.22–0.31)
Other	0.20***	0.37***	0.43***	0.36***	0.40***	0.31***
	(0.15–0.26)	(0.28–0.51)	(0.33–0.56)	(0.28–0.48)	(0.31–0.53)	(0.24–0.40)
**Reason for** OPD visit						
Sickness/Illness®						
Injury/Accident	1.06	1.08	0.89	1.21	-	1.35*
	(0.80–1.40)	(0.70–1.65)	(0.59–1.32)	(0.89–1.66)	-	(0.98–1.84)
Other	0.94	1.11	1.11	0.93	-	1.09
	(0.82–1.08)	(0.91–1.36)	(0.92–1.33)	(0.78–1.10)	-	(0.91–1.30)

**Constant**	0.26***	0.04***	0.11***	0.14***	0.13***	0.09***
	(0.17–0.39)	(0.02–0.08)	(0.06–0.20)	(0.08–0.24)	(0.08–0.22)	(0.05–0.16)

**Note:** p-values are represented as: ***<0.001; **<0.01; *<0.

**Table 5:** Variance partitioning coefficients (VPC) estimates from three level logistic regression for identification of negative experiences for six domains of patient experience among inpatient and outpatient service use among older adults aged 45 years or above, India, 2017-18.

	Inpatient			Outpatient		
	State	Village	Household	State	Village	Household
Waiting time	26.5	73.4	0.01	34.5	64.8	0.7
Respectful treatment	77.5	19.3	3.2	25.7	73.1	1.3
Clarity of explanation provided	57.4	42.5	0.1	39.0	57.3	3.7
Privacy during consultation	93.5	5.9	0.6	30.2	65.9	3.9
Provider of Choice	87.3	9.6	3.1	32.9	66.4	0.7
Cleanliness of facility	41.2	58.8	0.1	32.3	65.9	1.8

For outpatients, education, employment, and facility type predict negative ratings across most domains (Table 4). Higher education and private facility use correlate with fewer negative ratings, while the never-employed report more negatively on most domains. For instance, those with 10+ years of education have lower odds of negative ratings for respectful treatment (AOR: 0.58; 95 % CI: 0.44–0.77; p < 0.001) than the illiterate. Education, expenditure quintile, and facility type predict moderate ratings, showing a decline with higher education and expenditure. Variation in outpatient experiences is largely attributable to villages (Table 5).

## 4 Discussion

Our study yields five key findings. Firstly, over 70 % of individuals rated their experience across six domains as “Good” or “Very Good”, with the highest ratings for clarity of explanation among inpatients and cleanliness of facility for outpatient services. Negative experiences were less than 5% across all six domains. Secondly, positive experiences were consistently highest in private facilities and negative and moderate experiences were higher in public facilities on all six domains. Thirdly, cleanliness was a major issue for hospitalisation and waiting time for outpatient services in public facilities, while waiting time was the primary concern in private facilities for both services. Fourthly, socio-economic and demographic factors, particularly education, and income, were consistent predictors of negative experiences across multiple domains for inpatients and outpatients. Lastly, significant geographic variations in negative patient experiences were observed, with villages/CEBs attributing to the highest variation, especially in respectful treatment, provider choice, and privacy during consultation. Notably, higher-than-average negative experiences were noted in certain states for both inpatients and outpatients.

Existing evidence on patient satisfaction or experience in India diverges from our study in terms of regional specificity, investigated domains, age groups, and methodological approaches. However, notable parallels exist. For example, Persai et al. (2022) [19] found predominantly positive patient experiences in primary healthcare services across 13 districts, particularly in cleanliness and communication. Similarly, research in the Alipurduar district [23] highlighted positive feedback on responsiveness and information provision, with cleanliness as a key improvement area. A systematic review on tuberculosis treatment in India [24] also found satisfactory patient experiences, mirroring our findings.

### 4.1 Data related considerations

Our findings should be interpreted in the light of some data-related considerations. First, patient feedback are often influenced by factors outside the purview of clinics [[29], [30], [31]]. Second, given that older adults are the primary respondents, the influence of recall bias may be higher due to cognitive decline and limited literacy. Further, those in long-term care may be underrepresented here. Third, our study only considers six domains of patient experience out of the many more usually covered by hospital data or surveys designed specifically for capturing patient experience, such as medical/technical competence, shared decision-making, emotional support, ease of discharge and so on [8,[29], [30], [31], [32]]. Fourth, our study reflects patient experience in pre-COVID time (2018–19). This is important to note because the Government of India took substantial steps to improve the responsiveness of health system after the pandemic, such as investing in medical infrastructure in Tier II and III cities, increasing health insurance awareness, and larger use of technology in healthcare; that may have impacted patient experiences post lockdown [33,34].

It is imperative to acknowledge that certain limitations within our data, such as subjectivity and social desirability bias, as well as potential difficulties in understanding due to factors like age, education, or communication barriers, may lead to a tendency to report positive patient experiences or at least give moderate feedback, regardless of the actual encounters. This is evident from the findings that nearly one-third of the population notes a moderate patient experience on all six domains. Additionally, the exclusion of various dimensions of patient experience may result in an overrepresentation of positive reports. To counter such limitations in further studies, we suggest the use of mixed methods to account for subjectivity in patient experiences. The bias acquired in quantitative measures can be offset by a detailed account of personal experience of healthcare use.

### 4.2 High positive patient experience among older adults in India: artefact or reality?

On comparing our estimates with other global estimates; we found that India performs relatively better on some aspects such as waiting time with only 5% negative reportings contrasting with higher figures in countries like Australia (21%) and the UK (30%) [[35], [36], [37]]. Also, Indian older adults report good clarity of explanation from doctors, although slightly lower than major OECD countries. However, the reliability of patient experiences may be influenced by socio-economic factors, where lower socio-economic status could lead to lower expectations and a tendency to report positively due to social desirability. This phenomenon might manifest differently across countries or within India itself, depending on regional affluence.

Similar concerns about reporting heterogeneity exist regarding self reported healt measures. However, evidence indicates that self-reported health measures are reliable across socio-economic groups if reporting worsens with declining socio-economic status [[38], [39], [40]]. Similarly, we observe a negative association between socio-economic status and negative patient experiences across various domains. Also geographically, we observed that states such as Kerala, which is considered the most socio-economically developed state of India have much lower negative patient experience on five out of six domains compared to Bihar which is considered relatively underdeveloped. This suggests that the Patient-Reported Experience Measures (PREM) used in our study offer a credible reflection of patient experiences relative to socio-economic status.

### 4.3 Patient experience measurement in India and the way forward

The healthcare system in India is provider-centric, operating through individual providers or for-profit and nonprofit organizations that employ these providers [2]. With most payments being out-of-pocket, there is no third-party regulator for these assessments, leading to on-the-spot decisions. In poor health conditions, patients often resort to low-quality existing services at the provider's quoted price, primarily because they are unaware about their rights, thus reducing provider's incentive to focus on quality. However, further inquiry into this association is needed. Nevertheless, in such situations, sharing user experiences in healthcare serves as a quality assessment tool that empowers patients by informing them of basic quality domains. At the same time it can hold providers accountable without needing extensive medical knowledge.

Private healthcare institutions in India are increasingly relying on patient feedback and establishing separate patient experience departments for strengthening customer loyalty, brand building and increasing utilization [41]. However, current quality assessment of any public facilities in India happens through the health care facility accreditation system [42], the central tracking system [43] for district hospital performance or the Kayakalp scheme under the National Quality Assurance Programme [44], which rely on hospital characteristics such as infrastructure or cleanliness of the facility. Ministry of Health and Family Welfare has initiated the collection of patient feedback via the Mera-Aspataal portal (My hospital), which allows you to upload your experience of public hospital use via SMS, outbound dialling, or web survey in seven different languages [45].

Despite these developments, India lacks a national quality appraisal framework that puts the patient experience at center stage. This is particularly important for older adults, who will soon be the dominant healthcare seekers. Policy discussions should focus on evaluating elder-friendliness by assessing accessibility and mobility accommodations, integrating geriatric-centric assessments into protocols, and prioritizing staff training for sensitivity in elderly care.

In this scenario, estimates from large-scale surveys, such as this study, can identify broad areas needing immediate attention and guide quality appraisal. For example, public facility dissatisfaction often stems from waiting times, cleanliness, and privacy during consultations. Further, outlier case studies, such as those from Odisha, Sikkim, and Nagaland, where negative ratings for waiting times are below 2 %, can provide insights into effective strategies. For instance, Himachal Pradesh shows exceptionally low negative ratings (below 1 %, compared to an average of 4–5%) across all six domains for both inpatient and outpatient services, highlighting its robust healthcare system. The state boasts low out-of-pocket expenditures (around 50 %) [46] and widespread coverage through schemes like Ayushman Bharat Pradhan Mantri Jan Arogya Yojana and HIMCARE. Additionally, it enjoys surplus healthcare resources, with twice the required number of primary healthcare centres and 1.5 times the recommended number of community healthcare centres by the government of India [47]. These factors facilitate better service delivery by ensuring sufficient healthcare workforce, enabling improved time management and meaningful patient engagement, including better explanations, privacy, and respectful treatment. The reduced reliance on out-of-pocket expenses also minimizes dissatisfaction with services due to reduced concerns about costs relative to service quality. Additionally, states can learn from their own inpatient and outpatient departments. For example, in Tamil Nadu and Andhra Pradesh, cleanliness is less problematic in inpatient services but receives above-average negative ratings in outpatient services, indicating areas for targeted improvement.

Recent health metrics and institutional observations [1] consistently indicate that healthcare quality in India has substantial room for improvement. However, the positive assessment of healthcare quality in this study prompts critical reflection. It raises the question of whether this perceived quality genuinely signifies excellence or if it indicates that individuals' expectations are set relatively low. While we argue that PREM estimates are not mere artifacts, enhancing awareness about patient rights and healthcare quality will improve overall provider accountability and enhance the credibility of PREM-based measures. In this context, it is also imperative to evaluate healthcare quality using multiple methods that offer different perspectives [48]. Integrating these methods can mitigate their respective limitations and provide a more comprehensive assessment of quality.

## Data sharing statement

The data that support the findings of this study are openly available at https://www.iipsindia.ac.in/content/LASI-data.

## Funding

This study was supported by grant INV-002992 from the 10.13039/100000865Bill & Melinda Gates Foundation. The funder had no role in the design and conduct of the study; collection, management, analysis, and interpretation of the data; preparation, review, or approval of the manuscript; and decision to submit the manuscript for publication.

## Declaration of competing interest

The authors declare that they have no known competing financial interests or personal relationships that could have appeared to influence the work reported in this paper.
