# Supplementary material for: Experience of health care utilization for inpatient and outpatient services among older adults in India
Source: Public Health Pract (Oxf). 2024 Aug 22;8:100541. doi: 10.1016/j.puhip.2024.100541 (PMC11413678; doi:10.1016/j.puhip.2024.100541)
Supplement: Multimedia component 1 [file mmc1.docx]

**Experience of health care utilization for inpatient and outpatient services among older adults in India**

Supplementary materials

**Table S1:** Correlation of predictors with patient experience measures among older adults aged 45 years, India, 2017-18

|  | Inpatient | | | Outpatient | | |
| --- | --- | --- | --- | --- | --- | --- |
|  | Age | Education | Income | Age | Education | Income |
| Waiting time | 0.02 | -0.1 | -0.06 | 0.01 | -0.08 | -0.06 |
| p-value | 0.32 | 0 | 0 | 0.32 | 0.00 | 0.00 |
|  |  |  |  |  |  |  |
| Respectful treatment | 0 | -0.12 | -0.09 | 0.00 | -0.12 | -0.10 |
| p-value | 0.99 | 0 | 0 | 0.57 | 0.00 | 0.00 |
|  |  |  |  |  |  |  |
| Clarity of explanation provided | 0 | -0.1195 | -0.07 | 0.00 | -0.11 | -0.09 |
| p-value | 0.82 | 0 | 0 | 0.68 | 0.00 | 0.00 |
|  |  |  |  |  |  |  |
| Privacy during consultation | 0.006 | -0.1194 | -0.08 | 0.00 | -0.12 | -0.09 |
| p-value | 0.97 | 0 | 0 | 0.66 | 0.00 | 0.00 |
|  |  |  |  |  |  |  |
| Provider of Choice | 0 | -0.12 | -0.08 | 0.00 | -0.11 | -0.09 |
| p-value | 0.84 | 0 | 0 | 0.50 | 0.00 | 0.00 |
|  |  |  |  |  |  |  |
| Cleanliness of facility | 0.02 | -0.11 | -0.07 | 0.01 | -0.11 | -0.09 |
| p-value | 0.16 | 0 | 0 | 0.36 | 0.00 | 0.00 |

Table S2: Percentage of negative patient responses (95% CI) for their experience of inpatient services by characteristics of patients among older adults in India, 2017-18

|  | Waiting time | | Respectful treatment | | Clarity of explanation provided | | Privacy during consultation | | Provider of Choice | | Cleanliness of facility | |
| --- | --- | --- | --- | --- | --- | --- | --- | --- | --- | --- | --- | --- |
|  | % | 95% CI | % | 95% CI | % | 95% CI | % | 95% CI | % | 95% CI | % | 95% CI |
| **Overall** | 11.0 | (3.9-5.2) | 3.8 | (3.2-4.4) | 3.8 | (3.2-4.3) | 4.7 | (4-5.3) | 4.7 | (4.1-5.3) | 4.9 | (4.3-5.5) |
| **Age** |  |  |  |  |  |  |  |  |  |  |  |  |
| 45-54 | 4.4 | (3.3-5.6) | 2.2 | (1.4-3) | 3.0 | (2.1-4) | 4.1 | (3-5.2) | 4.4 | (3.2-5.5) | 4.0 | (3-5.1) |
| 55-64 | 4.0 | (2.9-5) | 3.2 | (2.3-4.2) | 3.6 | (2.6-4.6) | 4.5 | (3.4-5.6) | 4.6 | (3.5-5.8) | 4.8 | (3.7-6) |
| 65-74 | 4.0 | (2.8-5.1) | 4.4 | (3.2-5.6) | 3.7 | (2.6-4.8) | 4.9 | (3.6-6.1) | 4.0 | (2.9-5.2) | 4.2 | (3.1-5.4) |
| 75+ | 7.3 | (5.2-9.5) | 7.5 | (5.3-9.7) | 5.8 | (3.9-7.8) | 6.0 | (4-7.9) | 6.9 | (4.8-9) | 8.2 | (5.9-10.4) |
| **Gender** |  |  |  |  |  |  |  |  |  |  |  |  |
| Male | 5.8 | (4.8-6.8) | 4.2 | (3.4-5.1) | 4.1 | (3.2-4.9) | 4.8 | (3.9-5.7) | 4.8 | (3.9-5.7) | 5.3 | (4.4-6.3) |
| Female | 3.4 | (2.6-4.2) | 3.4 | (2.7-4.2) | 3.5 | (2.7-4.2) | 4.5 | (3.7-5.4) | 4.6 | (3.7-5.5) | 4.5 | (3.6-5.4) |
| **Marital Status** | |  |  |  |  |  |  |  |  |  |  |  |
| Currently married | 4.7 | (4-5.4) | 3.3 | (2.7-3.9) | 3.7 | (3.1-4.4) | 4.5 | (3.8-5.2) | 4.5 | (3.8-5.3) | 4.2 | (3.5-4.9) |
| Currently unmarried | 4.2 | (3-5.4) | 5.2 | (3.9-6.5) | 3.8 | (2.7-5) | 5.2 | (3.9-6.6) | 5.1 | (3.8-6.4) | 6.9 | (5.4-8.3) |
| **Education** |  |  |  |  |  |  |  |  |  |  |  |  |
| Illiterate | 3.8 | (3-4.7) | 4.2 | (3.3-5) | 3.7 | (2.8-4.5) | 5.5 | (4.5-6.5) | 4.7 | (3.8-5.3) | 5.6 | (4.6-6.6) |
| Less than 5 years | 6.3 | (4.3-8.3) | 6.3 | (4.3-8.3) | 5.6 | (3.7-7.5) | 5.1 | (3.3-7) | 5.0 | (3.2-6.4) | 4.4 | (2.7-6.1) |
| 5 to 9 years completed | 5.5 | (4.1-6.9) | 2.7 | (1.7-3.7) | 3.2 | (2.2-4.3) | 4.0 | (2.8-5.2) | 4.9 | (3.6-5.2) | 4.8 | (3.5-6.1) |
| 10 years or more completed | 4.0 | (2.6-5.4) | 2.0 | (1-3) | 3.2 | (1.9-4.5) | 2.2 | (1.1-3.2) | 4.1 | (2.6-9) | 3.0 | (1.7-4.2) |
| **Work status** |  |  |  |  |  |  |  |  |  |  |  |  |
| Currently working | 4.7 | (3.7-5.8) | 3.6 | (2.7-4.5) | 3.3 | (2.4-4.2) | 4.1 | (3.1-5.1) | 5.0 | (3.9-6.1) | 5.5 | (4.4-6.6) |
| Ever worked but currently not working | 4.8 | (3.8-5.8) | 4.3 | (3.3-5.2) | 4.4 | (3.4-5.3) | 5.3 | (4.2-6.3) | 4.6 | (3.6-5.6) | 4.6 | (3.6-5.6) |
| Never worked | 3.9 | (2.7-5) | 3.3 | (2.2-4.4) | 3.3 | (2.2-4.4) | 4.4 | (3.1-5.6) | 4.4 | (3.2-5.7) | 4.6 | (3.3-5.9) |
| **Expenditure Quintile** | |  |  |  |  |  |  |  |  |  |  |  |
| Lowest | 6.0 | (4-8) | 6.2 | (4.2-8.3) | 5.0 | (3.1-6.9) | 5.3 | (3.4-7.2) | 7.2 | (5-9.4) | 9.7 | (7.2-12.2) |
| Lowest | 5.3 | (3.6-7) | 3.6 | (2.2-5) | 3.7 | (2.3-5.1) | 4.5 | (3-6.1) | 5.0 | (3.4-6.7) | 5.8 | (4.1-7.6) |
| Middle | 4.8 | (3.3-6.3) | 5.3 | (3.7-6.9) | 3.3 | (2.1-4.6) | 7.7 | (5.8-9.5) | 4.5 | (3-5.9) | 5.1 | (3.6-6.7) |
| High | 4.1 | (2.8-5.4) | 4.4 | (3.1-5.7) | 5.5 | (4-7) | 4.2 | (2.9-5.4) | 5.1 | (3.7-6.5) | 4.5 | (3.1-5.8) |
| Highest | 3.7 | (2.7-4.7) | 1.5 | (0.9-2.2) | 2.3 | (1.5-3.1) | 3.0 | (2.1-3.9) | 3.3 | (2.3-4.2) | 2.4 | (1.6-3.3) |
| **Religion** |  |  |  |  |  |  |  |  |  |  |  |  |
| Hindu | 4.6 | (3.9-5.4) | 3.7 | (3-4.3) | 3.9 | (3.2-4.6) | 4.9 | (4.2-5.7) | 5.1 | (4.3-5.9) | 5.0 | (4.3-5.8) |
| Muslim | 3.5 | (1.9-5.1) | 2.8 | (1.4-4.2) | 2.8 | (1.4-4.3) | 3.3 | (1.7-4.8) | 2.3 | (1-3.6) | 3.5 | (1.9-5.1) |
| Others | 6.0 | (4.1-7.9) | 7.5 | (5.3-9.6) | 4.4 | (2.7-6) | 4.6 | (2.9-6.3) | 5.3 | (3.5-7.1) | 6.4 | (4.4-8.4) |
| **Caste** |  |  |  |  |  |  |  |  |  |  |  |  |
| Scheduled Castes | 3.9 | (2.5-5.3) | 4.2 | (2.8-5.7) | 4.2 | (2.7-5.6) | 4.5 | (3-6) | 5.8 | (4.1-7.4) | 7.6 | (5.7-9.5) |
| Scheduled Tribes | 4.9 | (3.3-6.6) | 4.2 | (2.6-5.7) | 5.0 | (3.3-6.7) | 8.8 | (6.6-11.1) | 3.7 | (2.2-5.2) | 5.7 | (3.9-7.6) |
| Other backward castes | 4.5 | (3.5-5.5) | 3.7 | (2.8-4.6) | 3.3 | (2.5-4.2) | 4.2 | (3.2-5.2) | 4.3 | (3.3-5.2) | 3.8 | (2.9-4.7) |
| Others | 5.0 | (3.8-6.2) | 3.5 | (2.5-4.6) | 3.9 | (2.8-4.9) | 4.5 | (3.4-5.7) | 4.9 | (3.7-6.1) | 4.5 | (3.3-5.6) |
| **Place of residence** | |  |  |  |  |  |  |  |  |  |  |  |
| Rural | 5.0 | (4.2-5.8) | 4.1 | (3.4-4.8) | 3.8 | (3.1-4.5) | 4.6 | (3.8-5.4) | 5.1 | (4.3-6) | 5.3 | (4.5-6.2) |
| Urban | 3.6 | (2.7-4.6) | 3.2 | (2.3-4.1) | 3.7 | (2.7-4.6) | 4.8 | (3.7-5.9) | 3.8 | (2.8-4.8) | 4.0 | (3-5) |
| **Facility type** | |  |  |  |  |  |  |  |  |  |  |  |
| Public | 6.2 | (5.1-7.3) | 5.7 | (4.7-6.8) | 5.0 | (4-6) | 7.8 | (6.5-9) | 7.5 | (6.3-8.8) | 9.5 | (8.1-10.8) |
| Private | 3.4 | (2.6-4.1) | 2.5 | (1.8-3.1) | 2.7 | (2-3.3) | 2.6 | (2-3.3) | 2.7 | (2.1-3.4) | 2.0 | (1.4-2.5) |
| Other | 9.5 | (4.1-14.9) | 8.1 | (3.1-13.2) | 11.5 | (5.7-17.4) | 9.7 | (4.2-15.1) | 10.8 | (5.1-16.5) | 9.7 | (4.2-15.1) |
| **Reason for hospitalisation** | | |  |  |  |  |  |  |  |  |  |  |
| Sickness/Illness | 4.2 | (3.6-4.9) | 3.6 | (3-4.2) | 3.7 | (3.1-4.3) | 4.4 | (3.8-5.1) | 4.6 | (3.9-5.2) | 5.0 | (4.3-5.7) |
| Injury/Accident | 7.6 | (5.1-10) | 5.6 | (3.5-7.7) | 4.1 | (2.3-6) | 7.0 | (4.7-9.4) | 5.3 | (3.2-7.4) | 4.2 | (2.4-6.1) |
| Other | 1.1 | (-3.5-5.8) | 1.1 | (-3.5-5.8) | 7.2 | (-4.1-18.6) | 0 | (-0.5-0.5) | 17.9 | (1.1-34.8) | 6.8 | (-4.2-17.9) |

Table S3: Percentage of negative patient responses (95% CI) for their experience of outpatient services by characteristics of patients among older adults in India, 2017-18

|  | Waiting time | | Respectful treatment | | Clarity of explanation provided | | Privacy during consultation | | Provider of Choice | | Cleanliness of facility | |
| --- | --- | --- | --- | --- | --- | --- | --- | --- | --- | --- | --- | --- |
|  | % | 95% CI | % | 95% CI | % | 95% CI | % | 95% CI | % | 95% CI | % | 95% CI |
| **Overall** | 4.6 | (4.4-4.8) | 2.1 | (2-2.3) | 2.9 | (2.7-3.1) | 3.3 | (3.1-3.4) | 3.1 | (2.9-3.3) | 2.7 | (2.6-2.9) |
| **Age** |  |  |  |  |  |  |  |  |  |  |  |  |
| 45-54 | 4.8 | (4.4-5.2) | 1.8 | (1.6-2.1) | 2.4 | (2.2-2.7) | 3.2 | (2.9-3.5) | 3.2 | (2.9-3.5) | 2.5 | (2.2-2.8) |
| 55-64 | 4.4 | (4-4.8) | 2.1 | (1.9-2.4) | 3 | (2.7-3.3) | 3.1 | (2.7-3.4) | 2.7 | (2.4-3) | 2.6 | (2.3-2.9) |
| 65-74 | 4.6 | (4.2-5.1) | 2.3 | (2-2.6) | 2.9 | (2.5-3.2) | 3.4 | (3-3.8) | 3.1 | (2.7-3.5) | 3 | (2.7-3.4) |
| 75+ | 4.3 | (3.6-5) | 2.6 | (2.1-3.1) | 4 | (3.4-4.7) | 3.6 | (2.9-4.2) | 3.9 | (3.3-4.6) | 3 | (2.4-3.5) |
| **Gender** |  |  |  |  |  |  |  |  |  |  |  |  |
| Male | 4.6 | (4.2-4.9) | 2 | (1.7-2.2) | 3 | (2.7-3.3) | 3.2 | (3-3.5) | 3.2 | (2.9-3.5) | 2.7 | (2.4-2.9) |
| Female | 4.6 | (4.3-4.9) | 2.3 | (2.1-2.5) | 2.8 | (2.6-3.1) | 3.3 | (3-3.5) | 3.1 | (2.8-3.3) | 2.8 | (2.5-3) |
| **Marital Status** | |  |  |  |  |  |  |  |  |  |  |  |
| Currently married | 4.6 | (4.3-4.8) | 1.9 | (1.7-2) | 2.7 | (2.5-2.9) | 3 | (2.8-3.2) | 3 | (2.8-3.2) | 2.5 | (2.3-2.7) |
| Currently unmarried | 4.6 | (4.2-5.1) | 2.9 | (2.6-3.3) | 3.4 | (3-3.7) | 3.9 | (3.5-4.3) | 3.4 | (3-3.8) | 3.2 | (2.9-3.6) |
| **Education** |  |  |  |  |  |  |  |  |  |  |  |  |
| Illiterate | 4.8 | (4.5-5.2) | 2.5 | (2.2-2.7) | 3.3 | (3-3.6) | 3.7 | (3.4-4) | 3.3 | (3-3.6) | 3.2 | (2.9-3.4) |
| Less than 5 years | 5.1 | (4.4-5.7) | 3 | (2.5-3.5) | 2.8 | (2.3-3.4) | 4.1 | (3.5-4.8) | 4 | (3.4-4.6) | 2.9 | (2.3-3.4) |
| 5 to 9 years completed | 4.5 | (4-4.9) | 1.5 | (1.2-1.8) | 3 | (2.6-3.4) | 2.9 | (2.6-3.3) | 3.2 | (2.8-3.6) | 2.2 | (1.9-2.6) |
| 10 years or more completed | 3.7 | (3.2-4.1) | 1.3 | (1-1.6) | 1.5 | (1.2-1.8) | 1.7 | (1.4-2.1) | 1.7 | (1.3-2) | 2 | (1.6-2.3) |
| **Work status** | |  |  |  |  |  |  |  |  |  |  |  |
| Currently working | 4.4 | (4.1-4.8) | 1.7 | (1.5-1.9) | 2.4 | (2.2-2.7) | 2.9 | (2.7-3.2) | 2.9 | (2.6-3.2) | 2.5 | (2.2-2.7) |
| Ever worked but currently not working | 4.9 | (4.4-5.3) | 2.7 | (2.3-3) | 3.8 | (3.4-4.2) | 3.9 | (3.5-4.3) | 3.7 | (3.3-4.1) | 2.9 | (2.6-3.2) |
| Never worked | 4.5 | (4.1-5) | 2.3 | (2-2.6) | 2.7 | (2.4-3) | 3.1 | (2.7-3.4) | 2.7 | (2.4-3) | 3 | (2.6-3.3) |
| **Expenditure Quintile** | |  |  |  |  |  |  |  |  |  |  |  |
| Lowest | 5.1 | (4.5-5.7) | 2.2 | (1.9-2.6) | 3.3 | (2.9-3.8) | 3.7 | (3.2-4.2) | 3.1 | (2.6-3.6) | 3.2 | (2.7-3.6) |
| Lowest | 4.4 | (3.9-4.9) | 2.4 | (2-2.8) | 3.1 | (2.7-3.5) | 3.5 | (3-3.9) | 3.2 | (2.8-3.6) | 2.8 | (2.4-3.1) |
| Middle | 4.5 | (4-5) | 2.3 | (2-2.7) | 2.7 | (2.3-3.1) | 3.1 | (2.7-3.6) | 2.9 | (2.5-3.3) | 3.3 | (2.9-3.7) |
| High | 4.5 | (4-5) | 2.1 | (1.8-2.4) | 2.5 | (2.2-2.9) | 3 | (2.6-3.4) | 3.2 | (2.8-3.6) | 2.5 | (2.1-2.9) |
| Highest | 4.4 | (3.9-4.9) | 1.6 | (1.3-1.9) | 2.9 | (2.5-3.3) | 3 | (2.6-3.4) | 3.1 | (2.7-3.5) | 1.9 | (1.6-2.2) |
| **Religion** |  |  |  |  |  |  |  |  |  |  |  |  |
| Hindu | 4.5 | (4.3-4.8) | 2.3 | (2.1-2.4) | 3 | (2.8-3.3) | 3.4 | (3.2-3.7) | 3.2 | (2.9-3.4) | 2.8 | (2.6-3) |
| Muslim | 5.3 | (4.7-6) | 1.8 | (1.4-2.2) | 2.5 | (2.1-3) | 2.7 | (2.2-3.1) | 2.8 | (2.3-3.3) | 2.2 | (1.8-2.6) |
| Others | 3.9 | (3.3-4.5) | 1.3 | (0.9-1.7) | 1.8 | (1.3-2.2) | 2.2 | (1.7-2.7) | 2.9 | (2.4-3.5) | 2.6 | (2.1-3.1) |
| **Caste** |  |  |  |  |  |  |  |  |  |  |  |  |
| Scheduled Castes | 4.5 | (4-5) | 2.2 | (1.8-2.6) | 3.3 | (2.9-3.8) | 3.8 | (3.3-4.3) | 3.5 | (3.1-4) | 3.6 | (3.1-4) |
| Scheduled Tribes | 5.1 | (4.4-5.8) | 2.1 | (1.7-2.6) | 4.1 | (3.4-4.7) | 3.3 | (2.8-3.9) | 3.3 | (2.7-3.9) | 2.8 | (2.2-3.3) |
| Other backward castes | 4.7 | (4.4-5.1) | 2.2 | (1.9-2.4) | 3 | (2.7-3.3) | 3.3 | (3-3.6) | 3.2 | (2.9-3.5) | 2.7 | (2.4-3) |
| Others | 4.3 | (3.9-4.7) | 2.1 | (1.8-2.3) | 2.1 | (1.9-2.4) | 2.8 | (2.4-3.1) | 2.6 | (2.3-3) | 2.2 | (1.9-2.4) |
| **Place of residence** | |  |  |  |  |  |  |  |  |  |  |  |
| Rural | 4.6 | (4.3-4.9) | 2.1 | (1.9-2.3) | 3.1 | (2.9-3.3) | 3.4 | (3.1-3.6) | 3.2 | (2.9-3.4) | 2.9 | (2.7-3.1) |
| Urban | 4.5 | (4.1-4.9) | 2.3 | (2.1-2.6) | 2.5 | (2.2-2.8) | 3 | (2.7-3.3) | 3 | (2.7-3.3) | 2.3 | (2-2.5) |
| **Facility type** | |  |  |  |  |  |  |  |  |  |  |  |
| Public | 9.4 | (8.8-10) | 4.1 | (3.7-4.5) | 5.3 | (4.9-5.8) | 6.3 | (5.8-6.8) | 6 | (5.5-6.4) | 6.1 | (5.7-6.6) |
| Private | 3.4 | (3.2-3.7) | 1.6 | (1.4-1.8) | 2.2 | (2-2.4) | 2.4 | (2.2-2.6) | 2.3 | (2.1-2.6) | 1.7 | (1.5-1.9) |
| Other | 1.8 | (1.4-2.2) | 1.3 | (1-1.7) | 2.2 | (1.7-2.6) | 2.1 | (1.6-2.5) | 1.8 | (1.4-2.3) | 1.9 | (1.4-2.3) |
| **Reason for hospitalisation** | | |  |  |  |  |  |  |  |  |  |  |
| Sickness/Illness | 4.5 | (4.2-4.7) | 2.2 | (2-2.3) | 2.8 | (2.6-3) | 3.3 | (3.1-3.6) | 3 | (2.8-3.2) | 2.7 | (2.5-2.9) |
| Injury/Accident | 5.6 | (4.2-7) | 2.2 | (1.4-3.1) | 2.6 | (1.7-3.6) | 3.5 | (2.4-4.6) | 4.3 | (3.1-5.5) | 3.9 | (2.8-5.1) |
| Other | 4.8 | (4.4-5.3) | 2 | (1.7-2.3) | 3.2 | (2.8-3.6) | 2.9 | (2.6-3.3) | 3.2 | (2.8-3.6) | 2.7 | (2.3-3.1) |

**Table S4:** Percentage of negative patient responses (95% CI) for their experience of inpatient services among older adults by states and union territories of India, 2017-18

|  | Waiting time | | Respectful treatment | | Clarity of explanation provided | | Privacy during consultation | | Provider of Choice | | Cleanliness of facility | |
| --- | --- | --- | --- | --- | --- | --- | --- | --- | --- | --- | --- | --- |
|  | % | 95% CI | % | 95% CI | % | 95% CI | % | 95% CI | % | 95% CI | % | 95% CI |
| India | 4.6 | (3.9-5.2) | 3.8 | (3.2-4.4) | 3.8 | (3.2-4.3) | 4.7 | (4-5.3) | 4.7 | (4.1-5.3) | 4.9 | (4.3-5.5) |
| **States** |  |  |  |  |  |  |  |  |  |  |  |  |
| Andhra Pradesh | 1.5 | 0-3.4) | 1.2 | (0-2.9) | 2.3 | (0.1-4.5) | 2.6 | (0.2-4.9) | 3.1 | (0.5-5.7) | 1.7 | (0-3.6) |
| Arunachal Pradesh | 2.8 | (0-6.9) | 2.1 | (0-5.8) | 2.1 | (0-5.8) | 2.1 | (0-5.8) | 8.3 | (1.3-15.2) | 8.3 | (1.3-15.2) |
| Assam | 0 | (0-0) | 1.6 | (0-4.5) | 1.4 | (0-4.1) | 3.6 | (0-7.8) | 4.5 | (0-9.1) | 2.5 | (0-5.9) |
| Bihar | 5.2 | (1.5-8.9) | 3.8 | (0.6-7) | 4.8 | (1.3-8.4) | 5.5 | (1.7-9.3) | 4.1 | (0.8-7.4) | 5.5 | (1.7-9.3) |
| Chhattisgarh | 5.7 | (0-11.9) | 4.2 | (0-9.7) | 2.4 | (0-6.6) | 2.1 | (0-5.9) | 4.2 | (0-9.6) | 1.5 | (0-4.8) |
| Goa | 7.5 | (2.5-12.6) | 4.9 | (0.8-9) | 1.0 | (0-3.0) | 2.9 | (0-6.1) | 1.8 | (0-4.3) | 11.2 | (5.2-17.2) |
| Gujarat | 4.0 | (0.8-7.1) | 1.7 | (0-3.9) | 2.4 | (0-4.9) | 5.7 | (2-9.5) | 0.9 | (0-2.6) | 0 | (0-0) |
| Haryana | 3.6 | (0.6-6.6) | 5.0 | (1.5-8.6) | 8.6 | (4.1-13.1) | 10.2 | (5.3-15) | 12.2 | (6.9-17.5) | 7.2 | (3-11.4) |
| Himachal Pradesh | 1.0 | (0-2.7) | 0.2 | (0-0.9) | 0 | (0-0) | 0 | (0-0) | 0.3 | (0-1.2) | 0.1 | (0-0.6) |
| Jharkhand | 0.4 | (0-1.6) | 1.9 | (0-4.5) | 0.7 | (0-2.4) | 0.7 | (0-2.4) | 0 | (0-0) | 1.1 | (0-3.1) |
| Karnataka | 8.1 | (3.9-12.2) | 3.7 | (0.8-6.6) | 4.4 | (1.3-7.6) | 5.0 | (1.6-8.3) | 4.3 | (1.2-7.4) | 2.1 | (0-4.4) |
| Kerala | 7.5 | (3.9-11.1) | 2.8 | (0.5-5.1) | 2.1 | (0.1-4.1) | 2.1 | (0.1-4.1) | 2.1 | (0.1-4.1) | 2.6 | (0.4-4.7) |
| Madhya Pradesh | 7.4 | (3.7-11.1) | 6.4 | (2.9-9.8) | 6.0 | (2.7-9.4) | 7.9 | (4.1-11.7) | 6.2 | (2.8-9.6) | 8.6 | (4.6-12.5) |
| Maharashtra | 2.3 | (0.6-4) | 3.3 | (1.3-5.3) | 1.4 | (0.1-2.7) | 2 | (0.4-3.6) | 2.9 | (1-4.8) | 1.9 | (0.4-3.5) |
| Manipur | 6.2 | (1.0-11.4) | 0.8 | (0-2.7) | 2.0 | (0-5.1) | 3.5 | (0-7.5) | 6.5 | (1.2-11.8) | 3.2 | (0-7.1) |
| Meghalaya | 0 | (0-0) | 0 | (0-0) | 0 | (0-0) | 0 | (0-0) | 0 | (0-0) | 0 | (0-0) |
| Mizoram | 0 | (0-0) | 0 | (0-0) | 0.8 | (0-3.2) | 0 | (0-0) | 0 | (0-0) | 1.3 | (0-4.3) |
| Nagaland | 0 | (0-0) | 0 | (0-0) | 0 | (0-0) | 0 | (0-0) | 0 | (0-0) | 2.8 | (0-8.2) |
| Odisha | 1.6 | (0-3.7) | 0.7 | (0-2.2) | 2.8 | (0-5.6) | 3.8 | (0.5-7.1) | 2.5 | (0-5.2) | 6.1 | (2-10.2) |
| Punjab | 10.7 | (5.4-16) | 6.3 | (2.2-10.5) | 7.4 | (2.9-11.8) | 8.2 | (3.5-12.9) | 6.9 | (2.6-11.2) | 6.3 | (2.2-10.5) |
| Rajasthan | 7.3 | (3.3-11.2) | 4.3 | (1.2-7.3) | 7.0 | (3.2-10.9) | 5.8 | (2.3-9.4) | 5.2 | (1.8-8.5) | 3.7 | (0.8-6.5) |
| Sikkim | 4.7 | (0-10.2) | 6.0 | (0-12.1) | 4.6 | (0-9.9) | 10.3 | (2.4-18.1) | 2.3 | (0-6.2) | 4.6 | (0-10) |
| Tamil Nadu | 2.0 | (0-4) | 2.6 | (0.3-4.9) | 1.4 | (0-3.2) | 1.5 | (0-3.3) | 4.7 | (1.6-7.8) | 2.7 | (0.3-5.1) |
| Telangana | 4.3 | (1.3-7.4) | 5.3 | (1.9-8.6) | 3.6 | (0.8-6.4) | 7.8 | (3.8-11.9) | 5.9 | (2.4-9.5) | 6.3 | (2.7-9.9) |
| Tripura | 4.5 | (0.6-8.3) | 3.4 | (0-6.8) | 4.8 | (0.9-8.8) | 7.9 | (2.9-12.9) | 5.4 | (1.2-9.6) | 12.3 | (6.2-18.4) |
| Uttar Pradesh | 4.8 | (1.9-7.8) | 6.4 | (3-9.7) | 6.6 | (3.2-10) | 7.0 | (3.5-10.4) | 8.5 | (4.7-12.3) | 9 | (5.1-12.9) |
| Uttarakhand | 3.2 | (0-7.2) | 1.2 | (0-3.8) | 1.8 | (0-4.9) | 3.8 | (0-8.3) | 5.8 | (0.4-11.2) | 5.4 | (0.2-10.7) |
| West Bengal | 3.9 | (1.4-6.4) | 4.6 | (1.8-7.3) | 3.5 | (1.1-5.8) | 4.6 | (1.9-7.4) | 6 | (2.9-9.1) | 11.4 | (7.3-15.5) |
| **Union Territories** |  |  |  |  |  |  |  |  |  |  |  |  |
| Andaman and Nicobar | 9.5 | (2.7-16.3) | 6.8 | (1-12.7) | 5.6 | (0.2-10.9) | 6.9 | (1-12.8) | 6.9 | (1-12.8) | 8.6 | (2.1-15.1) |
| Chandigarh | 3.1 | (0-7.6) | 0 | (0-0) | 0 | (0-0) | 7.2 | (0.7-13.8) | 2.6 | (0-6.7) | 2.6 | (0-6.7) |
| Dadra and Nagar Haveli | 3.6 | (0-7.8) | 0 | (0-0) | 1.2 | (0-3.7) | 1.2 | (0-3.7) | 1.3 | (0-3.9) | 0 | (0-0) |
| Daman and Diu | 0.9 | (0-2.9) | 0.5 | (0-2.1) | 0.5 | (0-2.1) | 0.5 | (0-2.1) | 0.5 | (0-2.1) | 0.5 | (0-2.1) |
| Delhi | 3.6 | (0-8.2) | 1.7 | (0-5.0) | 2.2 | (0-5.8) | 6.6 | (0.5-12.6) | 1.8 | (0-5.1) | 10.0 | (2.6-17.4) |
| Jammu and Kashmir | 18.9 | (9.9-27.9) | 7.4 | (1.4-13.4) | 6 | (0.6-11.5) | 7.3 | (1.3-13.2) | 4.6 | (0-9.5) | 5.0 | (0-10) |
| Lakshadweep | 0 | (0-0) | 0 | (0-0) | 0.8 | (0-2.9) | 0 | (0-0) | 2.3 | (0-5.6) | 0 | (0-0) |
| Puducherry | 0.3 | (0-1.7) | 0.5 | (0-2.3) | 0 | (0-0) | 0.5 | (0-2.3) | 1.6 | (0-4.6) | 0 | (0-0) |

**Table S5:** Percentage of negative patient responses (95% CI) for their experience of outpatient services among older adults by states and union territories of India, 2017-18

|  | Waiting time | | Respectful treatment | | Clarity of explanation provided | | Privacy during consultation | | Provider of Choice | | Cleanliness of facility | |
| --- | --- | --- | --- | --- | --- | --- | --- | --- | --- | --- | --- | --- |
|  | % | 95% CI | % | 95% CI | % | 95% CI | % | 95% CI | % | 95% CI | % | 95% CI |
| India | 4.6 | (4.4-4.8) | 2.1 | (2-2.3) | 2.9 | (2.7-3.1) | 3.3 | (3.1-3.4) | 3.1 | (2.9-3.3) | 2.7 | (2.6-2.9) |
| **States** |  |  |  |  |  |  |  |  |  |  |  |  |
| Andhra Pradesh | 3.8 | (2.8-4.9) | 1.4 | (0.7-2) | 1.9 | (1.2-2.7) | 2.9 | (2-3.8) | 3 | (2.1-3.9) | 3.4 | (2.4-4.4) |
| Arunachal Pradesh | 4.4 | (1.8-7) | 3 | (0.8-5.1) | 2.8 | (0.7-4.8) | 3 | (0.8-5.1) | 3.9 | (1.5-6.4) | 4 | (1.5-6.5) |
| Assam | 3.6 | (2.4-4.9) | 1.1 | (0.4-1.9) | 1.8 | (0.9-2.7) | 2.3 | (1.3-3.3) | 3 | (1.9-4.1) | 1.1 | (0.4-1.8) |
| Bihar | 3.4 | (2.7-4.1) | 1.2 | (0.7-1.6) | 1.4 | (0.9-1.9) | 2.1 | (1.5-2.7) | 1.8 | (1.3-2.3) | 2.5 | (1.9-3.1) |
| Chhattisgarh | 5.7 | (3.9-7.5) | 4 | (2.5-5.5) | 7.8 | (5.7-9.8) | 7.4 | (5.4-9.4) | 6.4 | (4.5-8.3) | 9.7 | (7.4-12) |
| Goa | 4.4 | (2.8-5.9) | 1.3 | (0.5-2.2) | 1.1 | (0.3-1.9) | 1.9 | (0.9-2.9) | 1.1 | (0.3-1.8) | 1.6 | (0.6-2.5) |
| Gujarat | 4.2 | (2.9-5.5) | 0.7 | (0.2-1.3) | 2.3 | (1.3-3.2) | 3.1 | (2-4.2) | 1.7 | (0.9-2.5) | 0.6 | (0.1-1.1) |
| Haryana | 5.1 | (3.7-6.4) | 4.5 | (3.2-5.9) | 7.3 | (5.7-9) | 7.5 | (5.8-9.1) | 7.8 | (6.1-9.5) | 3.1 | (2-4.1) |
| Himachal Pradesh | 4.7 | (3.2-6.3) | 0.5 | (0-1) | 0.7 | (0.1-1.3) | 1 | (0.3-1.8) | 0.3 | (0-0.8) | 0.2 | (0-0.6) |
| Jharkhand | 1.4 | (0.6-2.1) | 1.3 | (0.6-2) | 1.3 | (0.6-2.1) | 1.4 | (0.7-2.2) | 1.5 | (0.7-2.3) | 1.7 | (0.9-2.5) |
| Karnataka | 8.1 | (6.6-9.6) | 4.3 | (3.2-5.4) | 5.1 | (3.9-6.3) | 5.6 | (4.4-6.9) | 6.4 | (5.1-7.8) | 3.2 | (2.2-4.2) |
| Kerala | 10.9 | (9.3-12.6) | 1.5 | (0.8-2.2) | 0.9 | (0.4-1.4) | 2.6 | (1.8-3.5) | 2.5 | (1.7-3.4) | 2.1 | (1.3-2.9) |
| Madhya Pradesh | 9.3 | (7.5-11) | 7 | (5.4-8.5) | 9 | (7.2-10.7) | 7 | (5.4-8.5) | 5.8 | (4.4-7.2) | 6.6 | (5.1-8.1) |
| Maharashtra | 4 | (3.1-4.8) | 1.5 | (0.9-2) | 1.4 | (0.9-1.9) | 2.2 | (1.5-2.8) | 2 | (1.4-2.6) | 1.9 | (1.4-2.5) |
| Manipur | 7.2 | (5-9.5) | 0.9 | (0.1-1.7) | 1.7 | (0.6-2.8) | 1.3 | (0.3-2.4) | 2.4 | (1.1-3.7) | 1.9 | (0.7-3.1) |
| Meghalaya | 0 | (0-0) | 0 | (0-0) | 0 | (0-0) | 0 | (0-0) | 0 | (0-0) | 0 | (0-0) |
| Mizoram | 0 | (0-0) | 0 | (0-0) | 0.9 | (-0.4-2.2) | 0.6 | (-0.5-1.7) | 0.3 | (-0.4-1) | 0.6 | (-0.5-1.7) |
| Nagaland | 0.8 | (-1-2.8) | 0.8 | (-1-2.8) | 0 | (0-0) | 0.5 | (-0.9-1.9) | 0 | (0-0) | 0.9 | (-1-2.9) |
| Odisha | 1.8 | (1.1-2.6) | 1.3 | (0.7-1.9) | 1.7 | (0.9-2.4) | 2.4 | (1.6-3.2) | 2.8 | (1.9-3.7) | 3.1 | (2.1-4) |
| Punjab | 3.4 | (2.5-4.4) | 0.5 | (0.1-0.8) | 0.8 | (0.3-1.3) | 1.2 | (0.6-1.7) | 1.5 | (0.9-2.2) | 1.1 | (0.6-1.7) |
| Rajasthan | 6.6 | (5.2-7.9) | 2 | (1.2-2.8) | 3.1 | (2.2-4.1) | 3.1 | (2.1-4.1) | 2.5 | (1.7-3.4) | 2.4 | (1.5-3.2) |
| Sikkim | 0.8 | (-0.9-2.7) | 0.8 | (-0.9-2.7) | 4.1 | (0.2-8.1) | 0.8 | (-0.9-2.5) | 0.8 | (-0.9-2.5) | 3.8 | (0-7.6) |
| Tamil Nadu | 4.4 | (3.4-5.4) | 2.2 | (1.5-3) | 3.5 | (2.5-4.4) | 4.2 | (3.2-5.2) | 5.6 | (4.4-6.7) | 2.6 | (1.8-3.3) |
| Telangana | 2.9 | (1.9-3.9) | 1.7 | (1-2.5) | 2.1 | (1.2-2.9) | 2.7 | (1.8-3.7) | 2.9 | (2-3.9) | 3.1 | (2.1-4.1) |
| Tripura | 4 | (2-6) | 1.7 | (0.4-3) | 2.1 | (0.6-3.5) | 1.4 | (0.2-2.7) | 3.8 | (1.9-5.8) | 6.7 | (4.1-9.2) |
| Uttar Pradesh | 3.9 | (3.1-4.7) | 3 | (2.3-3.7) | 3.6 | (2.8-4.3) | 3.5 | (2.8-4.3) | 3.1 | (2.4-3.8) | 2.9 | (2.2-3.6) |
| Uttarakhand | 6.6 | (4.6-8.6) | 1.4 | (0.5-2.4) | 5.6 | (3.8-7.4) | 5 | (3.3-6.7) | 4.1 | (2.5-5.6) | 3.8 | (2.3-5.3) |
| West Bengal | 2.1 | (1.6-2.7) | 0.7 | (0.4-1) | 1.8 | (1.2-2.3) | 1.9 | (1.4-2.5) | 1.4 | (0.9-1.8) | 2.2 | (1.6-2.8) |
| **Union territories** | |  |  |  |  |  |  |  |  |  |  |  |
| Andaman and Nicobar | 30.7 | (25.3-36.1) | 1.9 | (0.3-3.5) | 0.9 | (-0.2-2) | 1.2 | (0-2.4) | 1.2 | (0-2.6) | 7.5 | (4.4-10.7) |
| Chandigarh | 2.3 | (1-3.6) | 1.9 | (0.8-3.1) | 1.5 | (0.5-2.5) | 2.4 | (1.1-3.7) | 1.6 | (0.5-2.6) | 2.7 | (1.3-4.1) |
| Dadra and Nagar Haveli | 3.7 | (1.4-5.9) | 1.8 | (0.2-3.4) | 3.3 | (1.1-5.4) | 2.4 | (0.5-4.2) | 3 | (1-5.1) | 0.3 | (-0.3-1) |
| Daman and Diu | 2.3 | (0.9-3.7) | 0.9 | (0-1.8) | 0.7 | (0-1.5) | 0.9 | (0-1.8) | 0.7 | (0-1.5) | 0.8 | (0-1.7) |
| Delhi | 6.3 | (4-8.6) | 2.5 | (1-4) | 1.5 | (0.3-2.7) | 2.4 | (0.9-3.9) | 1.8 | (0.5-3.1) | 4 | (2.1-5.9) |
| Jammu and Kashmir | 15.9 | (13.6-18.3) | 4 | (2.7-5.3) | 7.1 | (5.4-8.7) | 7.1 | (5.5-8.8) | 6 | (4.5-7.5) | 4.7 | (3.3-6.1) |
| Lakshadweep | 2.2 | (1-3.4) | 1.6 | (0.6-2.6) | 1.7 | (0.6-2.7) | 1.7 | (0.7-2.8) | 2.2 | (1-3.4) | 1.6 | (0.6-2.6) |
| Puducherry | 3.5 | (2.2-4.7) | 5.1 | (3.6-6.7) | 4.7 | (3.2-6.2) | 4.6 | (3.1-6.1) | 5 | (3.5-6.6) | 3.1 | (1.8-4.3) |

**Table S6:** Binary logistic regression results (Adjusted Odds Ratio) for **the** association between socio-economic characteristics of older adult outpatients with their moderate reporting inpatient service use experience **on** six domains of healthcare, India, 2017-18

| VARIABLES | Waiting time | | | Respectful treatment | | | Clarity of explanation provided | | Privacy during consultation | | | Provider of Choice | | | Cleanliness of facility |
| --- | --- | --- | --- | --- | --- | --- | --- | --- | --- | --- | --- | --- | --- | --- | --- |
| **Age** |  | | |  | | |  | |  | | |  | | |  |
| 45-54® |  | | |  | | |  | |  | | |  | | |  |
| 55-64 | 0.98 | | | 1.01 | | | 0.92 | | 0.98 | | | 0.96 | | | 0.87 |
|  | (0.80 - 1.19) | | | (0.82 - 1.23) | | | (0.75 - 1.12) | | (0.81 - 1.20) | | | (0.79 - 1.18) | | | (0.71 - 1.07) |
| 65-74 | 0.95 | | | 0.78** | | | 0.83 | | 0.96 | | | 0.87 | | | 0.98 |
|  | (0.76 - 1.18) | | | (0.62 - 0.99) | | | (0.66 - 1.05) | | (0.76 - 1.21) | | | (0.69 - 1.10) | | | (0.78 - 1.23) |
| 75+ | 0.92 | | | 0.88 | | | 0.81 | | 0.8 | | | 0.97 | | | 0.96 |
|  | (0.70 - 1.22) | | | (0.66 - 1.18) | | | (0.61 - 1.08) | | (0.60 - 1.07) | | | (0.74 - 1.28) | | | (0.72 - 1.30) |
| **Gender** |  | | |  | | |  | |  | | |  | | |  |
| Male® |  | | |  | | |  | |  | | |  | | |  |
| Female | 0.85 | | | 0.96 | | | 0.75*** | | 0.88 | | | 0.96 | | | 0.88 |
|  | (0.69 - 1.03) | | | (0.78 - 1.19) | | | (0.61 - 0.93) | | (0.72 - 1.07) | | | (0.79 - 1.17) | | | (0.71 - 1.08) |
| **Marital Status** | |  | | |  | | |  | | |  | | |  | |
| Currently married® | |  | | |  | | |  | | |  | | |  | |
| Currently unmarried | 1.02 | | | 0.91 | | | 1.11 | | 0.97 | | | 1.01 | | | 0.94 |
|  | (0.83 - 1.24) | | | (0.74 - 1.11) | | | (0.91 - 1.37) | | (0.80 - 1.18) | | | (0.83 - 1.23) | | | (0.77 - 1.16) |
| **Education** |  | | |  | | |  | |  | | |  | | |  |
| Illiterate® |  | | |  | | |  | |  | | |  | | |  |
| Less than 5 years | 1.14 | | | 1.07 | | | 0.81 | | 1.01 | | | 1.17 | | | 0.91 |
|  | (0.90 - 1.45) | | | (0.83 - 1.38) | | | (0.62 - 1.05) | | (0.79 - 1.29) | | | (0.91 - 1.50) | | | (0.69 - 1.18) |
| 5 to 9 years completed | 0.80** | | | 0.9 | | | 0.83* | | 0.86 | | | 0.81* | | | 0.73*** |
|  | (0.65 - 0.99) | | | (0.72 - 1.13) | | | (0.67 - 1.04) | | (0.69 - 1.05) | | | (0.65 - 1.00) | | | (0.58 - 0.93) |
| 10 years or more completed | 0.66*** | | | 0.75** | | | 0.60*** | | 0.74** | | | 0.67*** | | | 0.54*** |
|  | (0.50 - 0.87) | | | (0.57 - 0.98) | | | (0.45 - 0.80) | | (0.57 - 0.96) | | | (0.51 - 0.88) | | | (0.40 - 0.72) |
| **Work status** |  | | |  | | |  | |  | | |  | | |  |
| Currently working® | |  | | |  | | |  | | |  | | |  | |
| Ever worked but currently not working | 1.24** | | | 1.26** | | | 1.21* | | 1.20* | | | 1.20* | | | 1.13 |
|  | (1.03 - 1.50) | | | (1.04 - 1.54) | | | (1.00 - 1.48) | | (0.99 - 1.46) | | | (0.99 - 1.46) | | | (0.92 - 1.38) |
| Never worked | 1.35** | | | 1.2 | | | 1.39*** | | 1.40*** | | | 1.16 | | | 1.31** |
|  | (1.06 - 1.72) | | | (0.93 - 1.54) | | | (1.08 - 1.78) | | (1.10 - 1.80) | | | (0.91 - 1.48) | | | (1.03 - 1.68) |
| **Expenditure Quintile** | |  | | |  | | |  | | |  | | |  | |
| Lowest® |  | | |  | | |  | |  | | |  | | |  |
| Lowest | 1.11 | | | 0.99 | | | 1.07 | | 1.04 | | | 1.2 | | | 1.34* |
|  | (0.85 - 1.47) | | | (0.76 - 1.30) | | | (0.80 - 1.42) | | (0.78 - 1.38) | | | (0.91 - 1.58) | | | (0.99 - 1.82) |
| Middle | 1.04 | | | 0.78* | | | 1.21 | | 0.79 | | | 1 | | | 1.33* |
|  | (0.78 - 1.37) | | | (0.59 - 1.03) | | | (0.92 - 1.61) | | (0.60 - 1.05) | | | (0.76 - 1.33) | | | (0.98 - 1.80) |
| High | 1.17 | | | 0.83 | | | 1.05 | | 0.99 | | | 0.94 | | | 1.25 |
|  | (0.89 - 1.53) | | | (0.63 - 1.09) | | | (0.80 - 1.38) | | (0.76 - 1.29) | | | (0.72 - 1.24) | | | (0.92 - 1.70) |
| Highest | 0.94 | | | 0.72** | | | 1.08 | | 0.89 | | | 0.93 | | | 1.30* |
|  | (0.72 - 1.23) | | | (0.55 - 0.94) | | | (0.82 - 1.42) | | (0.68 - 1.17) | | | (0.71 - 1.21) | | | (0.96 - 1.74) |
| **Religion** |  | | |  | | |  | |  | | |  | | |  |
| Hindu® |  | | |  | | |  | |  | | |  | | |  |
| Muslim | 0.87 | | | 1.03 | | | 1.04 | | 1.07 | | | 0.96 | | | 0.79 |
|  | (0.65 - 1.17) | | | (0.77 - 1.40) | | | (0.76 - 1.42) | | (0.80 - 1.43) | | | (0.71 - 1.30) | | | (0.58 - 1.09) |
| Others | 0.89 | | | 0.84 | | | 0.93 | | 0.81 | | | 0.95 | | | 1.03 |
|  | (0.66 - 1.21) | | | (0.60 - 1.17) | | | (0.65 - 1.32) | | (0.58 - 1.14) | | | (0.68 - 1.32) | | | (0.73 - 1.45) |
| **Caste** |  | | |  | | |  | |  | | |  | | |  |
| Scheduled Castes® | |  | | |  | | |  | | |  | | |  | |
| Scheduled Tribes | 1.05 | | | 0.97 | | | 1 | | 0.74 | | | 0.99 | | | 0.75 |
|  | (0.76 - 1.46) | | | (0.71 - 1.34) | | | (0.70 - 1.43) | | (0.51 - 1.07) | | | (0.71 - 1.38) | | | (0.53 - 1.08) |
| Other backward castes | 1.1 | | | 0.85 | | | 1.03 | | 0.75** | | | 0.94 | | | 0.98 |
|  | (0.88 - 1.37) | | | (0.68 - 1.08) | | | (0.81 - 1.31) | | (0.60 - 0.95) | | | (0.75 - 1.19) | | | (0.77 - 1.24) |
| Others | 0.89 | | | 1.01 | | | 0.84 | | 0.75** | | | 0.94 | | | 1.04 |
|  | (0.69 - 1.15) | | | (0.79 - 1.30) | | | (0.64 - 1.10) | | (0.58 - 0.97) | | | (0.74 - 1.21) | | | (0.80 - 1.34) |
| **Place of residence** | |  | | |  | | |  | | |  | | |  | |
| Rural® |  | | |  | | |  | |  | | |  | | |  |
| Urban | 0.94 | | | 0.80** | | | 0.83* | | 0.79** | | | 0.87 | | | 0.84* |
|  | (0.77 - 1.15) | | | (0.65 - 0.99) | | | (0.68 - 1.02) | | (0.65 - 0.95) | | | (0.71 - 1.06) | | | (0.68 - 1.03) |
| **Facility type** | |  | | |  | | |  | | |  | | |  | |
| Public® |  | | |  | | |  | |  | | |  | | |  |
| Private | 0.56*** | | | 0.54*** | | | 0.54*** | | 0.58*** | | | 0.47*** | | | 0.50*** |
|  | (0.47 - 0.67) | | | (0.45 - 0.64) | | | (0.45 - 0.64) | | (0.49 - 0.69) | | | (0.40 - 0.56) | | | (0.42 - 0.60) |
| Other | 0.78 | | | 0.97 | | | 0.79 | | 0.8 | | | 0.79 | | | 0.7 |
|  | (0.48 - 1.26) | | | (0.61 - 1.53) | | | (0.48 - 1.29) | | (0.49 - 1.29) | | | (0.50 - 1.27) | | | (0.43 - 1.15) |
| **Reason for hospitalisation** | | |  | | |  | | | |  | | |  | | |
| Sickness/Illness® | |  | | |  | | |  | | |  | | |  | |
| Injury/Accident | 0.99 | | | 0.98 | | | 1.03 | | 1.08 | | | 1.05 | | | 0.99 |
|  | (0.77 - 1.26) | | | (0.76 - 1.26) | | | (0.79 - 1.35) | | (0.84 - 1.40) | | | (0.82 - 1.35) | | | (0.76 - 1.29) |
| Other | 0.88 | | | 0.58 | | | 0.38 | | 0.33 | | | 0.66 | | | 1.64 |
|  | (0.34 - 2.26) | | | (0.15 - 2.24) | | | (0.10 - 1.39) | | (0.07 - 1.45) | | | (0.22 - 2.01) | | | (0.56 - 4.80) |
|  |  | | |  | | |  | |  | | |  | | |  |
| **Constant** | 0.7 | | | 0.55* | | | 0.37*** | | 0.45** | | | 0.41** | | | 0.36*** |
|  | (0.37 - 1.31) | | | (0.29 - 1.05) | | | (0.18 - 0.76) | | (0.21 - 0.94) | | | (0.21 - 0.81) | | | (0.18 - 0.73) |

**Table S7:** Binary logistic regression results (Adjusted Odds Ratio) for the association between socio-economic characteristics of older adult outpatients with their moderate reporting outpatient service use experience **on** six domains of healthcare, India, 2017-18

| VARIABLES | Waiting time | Respectful treatment | Clarity of explanation provided | Privacy during consultation | Provider of Choice | Cleanliness of facility |
| --- | --- | --- | --- | --- | --- | --- |
| **Age** |  |  |  |  |  |  |
| 45-54® |  |  |  |  |  |  |
| 55-64 | 0.99 | 0.97 | 0.97 | 0.98 | 0.99 | 1.01 |
|  | (0.92 - 1.05) | (0.90 - 1.04) | (0.91 - 1.04) | (0.92 - 1.05) | (0.93 - 1.06) | (0.94 - 1.08) |
| 65-74 | 0.96 | 0.92* | 0.95 | 0.94 | 1.02 | 0.90*** |
|  | (0.89 - 1.04) | (0.85 - 1.00) | (0.88 - 1.03) | (0.87 - 1.02) | (0.94 - 1.10) | (0.83 - 0.97) |
| 75+ | 1.04 | 1 | 0.98 | 0.99 | 1.08 | 0.98 |
|  | (0.93 - 1.15) | (0.90 - 1.12) | (0.89 - 1.09) | (0.89 - 1.10) | (0.97 - 1.20) | (0.88 - 1.09) |
| **Gender** |  |  |  |  |  |  |
| Male® |  |  |  |  |  |  |
| Female | 0.92** | 0.97 | 0.94* | 0.95 | 0.93** | 0.91** |
|  | (0.86 - 0.98) | (0.90 - 1.04) | (0.88 - 1.01) | (0.89 - 1.02) | (0.87 - 0.99) | (0.85 - 0.98) |
| **Marital Status** | |  |  |  |  |  |
| Currently married® | |  |  |  |  |  |
| Currently unmarried | 1 | 1.04 | 1.04 | 1.02 | 1.07* | 1.13*** |
|  | (0.94 - 1.07) | (0.97 - 1.12) | (0.97 - 1.12) | (0.95 - 1.09) | (0.99 - 1.14) | (1.05 - 1.21) |
| **Education** |  |  |  |  |  |  |
| Illiterate® |  |  |  |  |  |  |
| Less than 5 years | 0.91** | 0.92* | 0.94 | 0.93 | 0.93 | 0.96 |
|  | (0.83 - 1.00) | (0.83 - 1.01) | (0.86 - 1.03) | (0.85 - 1.02) | (0.84 - 1.02) | (0.88 - 1.06) |
| 5 to 9 years completed | 0.87*** | 0.87*** | 0.82*** | 0.85*** | 0.83*** | 0.83*** |
|  | (0.80 - 0.94) | (0.80 - 0.94) | (0.76 - 0.89) | (0.79 - 0.92) | (0.77 - 0.91) | (0.77 - 0.90) |
| 10 years or more completed | 0.72*** | 0.68*** | 0.69*** | 0.66*** | 0.72*** | 0.74*** |
|  | (0.65 - 0.79) | (0.61 - 0.75) | (0.63 - 0.77) | (0.60 - 0.73) | (0.65 - 0.79) | (0.67 - 0.82) |
| **Work status** | |  |  |  |  |  |
| Currently working® | |  |  |  |  |  |
| Ever worked but currently not working | 1 | 1.02 | 1.02 | 1.03 | 0.99 | 0.99 |
|  | (0.93 - 1.08) | (0.95 - 1.10) | (0.95 - 1.10) | (0.96 - 1.11) | (0.92 - 1.07) | (0.92 - 1.07) |
| Never worked | 1.11** | 1.03 | 1.10** | 1.13*** | 1.11** | 1.06 |
|  | (1.02 - 1.21) | (0.95 - 1.12) | (1.01 - 1.20) | (1.03 - 1.22) | (1.02 - 1.21) | (0.97 - 1.16) |
| **Expenditure Quintile** | |  |  |  |  |  |
| Lowest® |  |  |  |  |  |  |
| Lowest | 0.93 | 0.93 | 0.98 | 0.98 | 0.97 | 0.94 |
|  | (0.85 - 1.02) | (0.84 - 1.02) | (0.90 - 1.08) | (0.89 - 1.07) | (0.88 - 1.06) | (0.86 - 1.03) |
| Middle | 0.84*** | 0.83*** | 0.90** | 0.86*** | 0.92* | 0.84*** |
|  | (0.76 - 0.93) | (0.75 - 0.92) | (0.81 - 1.00) | (0.78 - 0.95) | (0.83 - 1.01) | (0.76 - 0.93) |
| High | 0.87*** | 0.84*** | 0.85*** | 0.86*** | 0.84*** | 0.80*** |
|  | (0.79 - 0.96) | (0.75 - 0.93) | (0.77 - 0.94) | (0.78 - 0.95) | (0.76 - 0.93) | (0.72 - 0.88) |
| Highest | 0.78*** | 0.76*** | 0.80*** | 0.78*** | 0.82*** | 0.79*** |
|  | (0.70 - 0.87) | (0.68 - 0.85) | (0.72 - 0.89) | (0.70 - 0.87) | (0.73 - 0.91) | (0.71 - 0.88) |
| **Religion** |  |  |  |  |  |  |
| Hindu® |  |  |  |  |  |  |
| Muslim | 0.95 | 0.92 | 0.99 | 0.95 | 0.99 | 0.94 |
|  | (0.84 - 1.07) | (0.81 - 1.04) | (0.88 - 1.12) | (0.84 - 1.06) | (0.88 - 1.12) | (0.83 - 1.07) |
| Others | 1 | 1.06 | 1.04 | 1.01 | 1.02 | 1.06 |
|  | (0.88 - 1.14) | (0.93 - 1.22) | (0.91 - 1.19) | (0.88 - 1.16) | (0.88 - 1.17) | (0.92 - 1.23) |
| **Caste** |  |  |  |  |  |  |
| Scheduled Castes® | |  |  |  |  |  |
| Scheduled Tribes | 1.02 | 1.01 | 1.04 | 1.06 | 1.08 | 1.03 |
|  | (0.88 - 1.17) | (0.88 - 1.16) | (0.90 - 1.20) | (0.92 - 1.22) | (0.94 - 1.24) | (0.89 - 1.19) |
| Other backward castes | 0.91** | 0.93 | 0.91** | 0.93* | 0.96 | 0.89** |
|  | (0.83 - 0.99) | (0.85 - 1.02) | (0.83 - 0.99) | (0.86 - 1.01) | (0.88 - 1.05) | (0.81 - 0.97) |
| Others | 0.98 | 0.92 | 0.90** | 0.93 | 0.96 | 0.93 |
|  | (0.88 - 1.08) | (0.83 - 1.02) | (0.81 - 0.99) | (0.85 - 1.02) | (0.87 - 1.06) | (0.83 - 1.03) |
| **Place of residence** | |  |  |  |  |  |
| Rural® |  |  |  |  |  |  |
| Urban | 0.90** | 0.88*** | 0.85*** | 0.85*** | 0.79*** | 0.85*** |
|  | (0.82 - 0.99) | (0.80 - 0.97) | (0.77 - 0.93) | (0.78 - 0.93) | (0.72 - 0.87) | (0.77 - 0.94) |
| **Facility type** | |  |  |  |  |  |
| Public® |  |  |  |  |  |  |
| Private | 0.66*** | 0.55*** | 0.61*** | 0.58*** | 0.56*** | 0.55*** |
|  | (0.62 - 0.71) | (0.51 - 0.59) | (0.57 - 0.66) | (0.54 - 0.62) | (0.52 - 0.60) | (0.51 - 0.59) |
| Other | 0.67*** | 0.75*** | 0.85*** | 0.77*** | 0.80*** | 0.85*** |
|  | (0.60 - 0.74) | (0.68 - 0.84) | (0.76 - 0.95) | (0.69 - 0.86) | (0.72 - 0.89) | (0.77 - 0.95) |
| **Reason for hospitalisation** | |  |  |  |  |  |
| Sickness/Illness® | |  |  |  |  |  |
| Injury/Accident | 1.07 | 1.04 | 1.01 | 0.99 | 0.96 | 0.99 |
|  | (0.93 - 1.23) | (0.89 - 1.21) | (0.87 - 1.17) | (0.85 - 1.15) | (0.83 - 1.12) | (0.85 - 1.15) |
| Other | 0.93* | 0.86*** | 0.85*** | 0.84*** | 0.89*** | 0.91** |
|  | (0.86 - 1.01) | (0.80 - 0.93) | (0.78 - 0.92) | (0.78 - 0.91) | (0.82 - 0.96) | (0.84 - 0.99) |
|  |  |  |  |  |  |  |
| **Constant** | 1.02 | 0.78* | 0.48*** | 0.57*** | 0.58*** | 0.60*** |
|  | (0.78 - 1.32) | (0.60 - 1.03) | (0.36 - 0.63) | (0.43 - 0.74) | (0.43 - 0.77) | (0.44 - 0.82) |

**Figure S1:** Percentage of older adults who reported a ‘bad’ or a ‘very bad’ experience of inpatient and outpatient service use by type of facility in rural and urban India, 2017-18


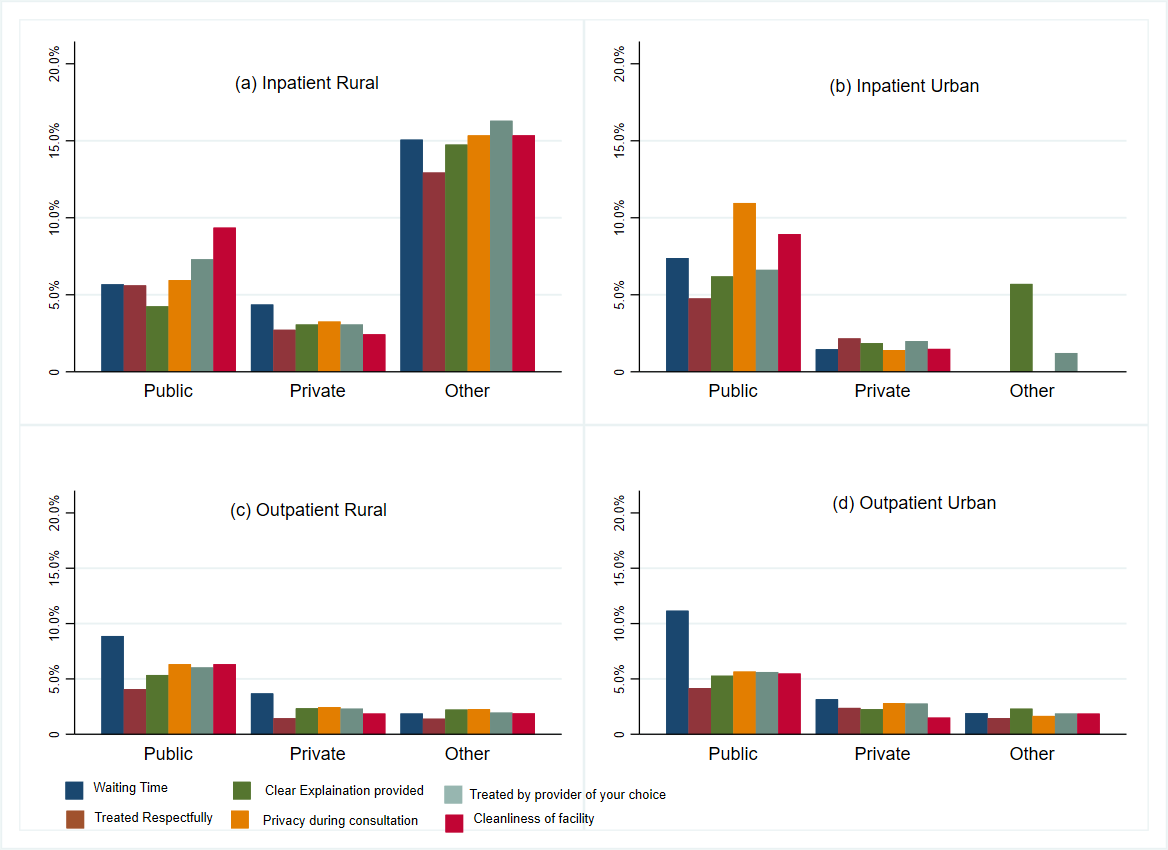


**Supplementary text 1**

**RESEARCH IN CONTEXT**

**Evidence before this study**

We searched PubMed and Web of Sciences to identify empirical studies that explored experience of people using healthcare facilities (outpatient or inpatient) and their satisfaction with the service in India. We searched for articles that had the combination of keywords ("Patient experiences" OR "Patient satisfaction" OR "Patient related experience measures" OR "Patient related outcome measures" OR "PREM" OR "PROM" ) AND ("India" OR "Indian") in their title or abstract. The search yielded 507 results (353 in PUBMED and 152 in Web of Sciences) of which we shortlisted 45 articles after titular screening and deleting duplicates. Majority of the included articles studied patient satisfaction (n=35), were disease specific (n=27), hospital based (n=22) or sub-national (n=14), of which only one referred to older patients. Further, while a few studies drew samples from various regions of India, their sample sizes ranged from 41 to 1046, making them less representative of the entire population.

**Added value of this study**

Our study provides the first systematic and nationally representative assessment of patient experiences among older adults in India. The study is comprehensive in that we assessed the experience of older adults on six major domains of patient interaction- waiting time, cleanliness, respectful treatment, getting treated by provider of choice, privacy during consultation, and clarity of explanation provided for both inpatient and outpatient visits across all states and union territories of India. The assessment provides the first ever insight on patient experiences at all India levels and a comparative assessment across socio-economic categories and geographical regions. Overall, the findings indicate that older adults in India generally have positive experiences with healthcare services. Among the reported negative experiences (less than 5%), most concerns revolve around waiting times and facility cleanliness. Additionally, a higher proportion of negative experiences are associated with public healthcare facilities compared to private ones.

**Implications of all the available evidence**

Insights obtained from patient experiences offer critical observations regarding the current status of our healthcare system. Specifically, the Indian public healthcare system, which sees lower utilization when compared to private healthcare providers, can utilize these patient experiences to identify areas in need of improvement and potentially broaden its user base.
